# The host cell secretory pathway mediates the export of *Leishmania* virulence factors out of the parasitophorous vacuole

**DOI:** 10.1371/journal.ppat.1007982

**Published:** 2019-07-29

**Authors:** Guillermo Arango Duque, Armando Jardim, Étienne Gagnon, Mitsunori Fukuda, Albert Descoteaux

**Affiliations:** 1 INRS, Centre Armand-Frappier Santé Biotechnologie, Laval, Québec, Canada; 2 Institute of Parasitology, McGill University, Ste-Anne-de-Bellevue, Québec, Canada; 3 Département de Microbiologie et Immunologie, Institut de Recherche en Immunologie et Cancer, Université de Montréal, Montréal, Québec, Canada; 4 Department of Integrative Life Sciences, Graduate School of Life Sciences, Tohoku University, Sendai, Miyagi, Japan; University of São Paulo FMRP/USP, BRAZIL

## Abstract

To colonize phagocytes, *Leishmania* subverts microbicidal processes through components of its surface coat that include lipophosphoglycan and the GP63 metalloprotease. How these virulence glycoconjugates are shed, exit the parasitophorous vacuole (PV), and traffic within host cells is poorly understood. Here, we show that lipophosphoglycan and GP63 are released from the parasite surface following phagocytosis and redistribute to the endoplasmic reticulum (ER) of macrophages. Pharmacological disruption of the trafficking between the ER and the Golgi hindered the exit of these molecules from the PV and dampened the cleavage of host proteins by GP63. Silencing by RNA interference of the soluble *N*-ethylmaleimide-sensitive-factor attachment protein receptors Sec22b and syntaxin-5, which regulate ER-Golgi trafficking, identified these host proteins as components of the machinery that mediates the spreading of *Leishmania* effectors within host cells. Our findings unveil a mechanism whereby a vacuolar pathogen takes advantage of the host cell's secretory pathway to promote egress of virulence factors beyond the PV.

## Author summary

*Leishmania* promastigotes are internalized by phagocytes into a highly modified phagosome that promotes parasite growth and differentiation into the amastigote form. To survive in the phagosome, *Leishmania* employs surface-bound glycoconjugates such as the GP63 metalloprotease and lipophosphoglycan to subvert the phagosome’s microbicidal potential. In particular, GP63 cleaves host cell vesicle fusion molecules that regulate phagosomal processes ranging from antigen cross-presentation to cytokine secretion. Unlike apicomplexan parasites and bacteria, *Leishmania* does not inject its virulence-associated glycoconjugates across the phagosome membrane. We found that post-phagocytosis, *Leishmania* co-opts the host cell secretory pathway to promote the egress of its virulence factors out of the phagosome. Importantly, chemical and genetic inhibition of endoplasmic reticulum (ER) to Golgi transport hindered the redistribution of GP63 and lipophosphoglycan, thereby impeding the cleavage of GP63 target Synaptotagmin XI. Notably, knockdown ER/ERGIC-resident membrane fusion regulators Sec22b and syntaxin-5 revealed that these host molecules were essential to the phagosomal egress of *Leishmania* virulence factors. These findings provide new insight into how *Leishmania* sabotages the host cell endomembrane system for its own benefit.

## Introduction

The protozoan parasite *Leishmania* causes a spectrum of human diseases known as the leishmaniases [1]. Infectious insect-stage promastigotes are inoculated in mammals upon the blood meal of the vector and are internalized by phagocytes where they differentiate into mammalian-stage amastigotes [2]. In contrast to amastigotes, promastigotes exist only transiently within mammals. To avoid destruction within phagocytes, promastigotes derail phagolysosome biogenesis and function, shut down microbicidal pathways, and sabotage immune processes [3–10]. Subversion of these host defence processes is achieved to a large extent through the action of the two major components of the *Leishmania* promastigote surface coat: the metalloprotease GP63 and lipophosphoglycan (LPG), a polymer of repeating Galβ1,4Manα1-PO_4_ units attached to the promastigote surface via a glycosylphosphatidylinositol anchor [11, 12]. Whereas GP63 acts by cleaving a set of host cell molecules that includes components of signaling cascades, transcription factors, and regulators of membrane fusion [13, 14], LPG inserts into lipid microdomains of host cell membranes, causing the disorganization of these structures [15, 16]. One direct consequence of LPG-mediated lipid microdomain disorganization is the inhibition of phagolysosome biogenesis [17], which is characterized by the exclusion of the v-ATPase and of the NADPH oxidase from the phagosome membrane [18, 19]. As LPG and GP63 are highly down-modulated or absent in mammalian-stage amastigotes [20, 21], their role is to create an environment permissive for the establishment of infection and propitious to promastigote-to-amastigote differentiation within phagocytes.

To exert their action, LPG and GP63 redistribute within infected cells but little is known with respect to their exact sub-cellular localization [3, 7, 22, 23]. In this regard, both LPG and GP63 associate with host membrane lipid microdomains and GP63 was shown to localize to the perinuclear area and the nuclear envelope of host cells [8, 24]. In addition, the mechanism by which LPG and GP63 are exported beyond the PV remains to be identified. This is a particularly intriguing issue since in contrast to bacterial pathogens or apicomplexan parasites [25, 26], *Leishmania* has no known specialized machinery aimed at injecting effectors inside the host cells. Instead, *Leishmania* promastigotes release exosomes containing hundreds of proteins including GP63 and it has been proposed that these nanovesicles contribute to pathogenesis by mediating the delivery of *Leishmania* molecules into host cells [27, 28]. However, how virulence factors and exosomes produced by *Leishmania* traffic beyond the PV remains to be elucidated. The observation that *Leishmania*-harboring PVs continuously interact with the secretory pathway [29] suggests a potential mechanism for the spread of *Leishmania*-derived material within host cells. Here, we report that GP63 and LPG are shed from the parasite surface upon phagocytosis and redistribute in the ER of infected cells via a mechanism involving vesicular trafficking between the PV and the host cell secretory pathway.

## Results

### Components of the *Leishmania* surface coat redistribute within phagocytes

**Fig 1:**
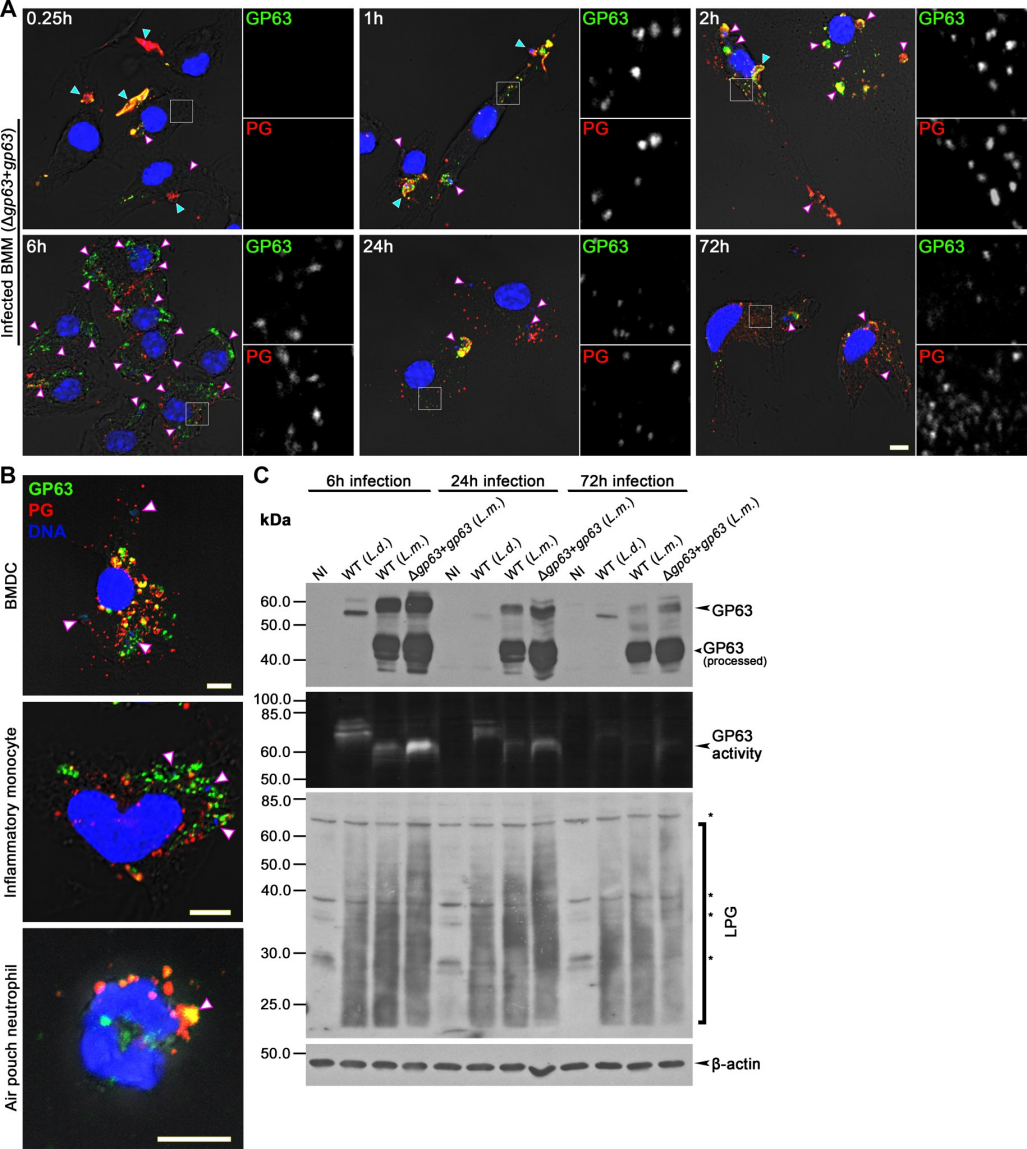
GP63 and PGs traffic within infected cells. **(A)** BMM were infected with opsonized *L*. *major* Δ*gp63*+*gp63* metacyclic promastigotes and the distribution of GP63 (green) and PGs (red) was monitored via confocal microscopy over a period of 0.25–72 h. 5X-enlarged channel-split insets of representative cytoplasmic regions are shown. **(B)** BMDC, inflammatory monocytes and air pouch neutrophils were infected for 6 h with *L*. *major* Δ*gp63*+*gp63* metacyclic promastigotes. Redistribution of GP63 (green) and PGs (red) in the cytoplasm of infected cells was observed via immunofluorescence. DNA is shown in blue. Representative images of at least three experiments are shown. In panels (A) and (B), white and cyan arrowheads denote internalized and non-internalized parasites, respectively. Bar, 5 μm. **(C)** The levels of GP63 and PGs in lysates from *L*. *major*- or *L*. *donovani*-infected BMM were assessed by Western blot analysis. NI, non-infected. (*) indicate non-specific bands of macrophage origin (see NI lanes). The activity of GP63 was assayed via gelatin zymography. Representative blots of at least two experiments are shown.

To investigate the mechanism by which virulence factors that constitute the surface coat of *Leishmania* are transported out of the PV and reach locations within host cells, we infected bone marrow-derived macrophages (BMM) with serum-opsonized *L*. *major* Δ*gp63*+*gp63* metacyclic promastigotes and we assessed by confocal immunofluorescence microscopy the fate of GP63 and phosphoglycans (PGs), a family of Gal-Man-PO_4_-containing glycoconjugates that comprises predominantly the GPI-anchored molecules LPG and proteophosphoglycan (PPG) [12, 30]. Infections were performed with the complemented Δ*gp63* mutant (Δ*gp63*+*gp63*), which displays the features of the wild-type parental strain (S8 Fig) and is better suited for comparative experiments involving the Δ*gp63* mutant. Fig 1A shows that GP63 and PGs dispersed in a time-dependent fashion from the PV harboring parasites to the cytoplasm of infected macrophages as early as 15 min upon the initiation of phagocytosis and remained detectable in infected cells over 72 h. We noticed that by 6 h post-phagocytosis, most of these components of the promastigote surface coat were shed from the parasites and were redistributed beyond the PV (Fig 1A). The redistribution pattern and kinetics for PGs and GP63 in BMM infected with metacyclic *L*. *braziliensis*, *L*. *chagasi*, and *L*. *donovani* promastigotes were similar to that observed in macrophages infected with *L*. *major* promastigotes (S1A and S1B Fig). Release and redistribution of these molecules also occurred in other phagocytes known to internalize *Leishmania*, including dendritic cells, inflammatory monocytes, and neutrophils (Fig 1B). Collectively, these data suggest a general virulence strategy triggered upon internalization of metacyclic promastigotes by phagocytic cells.

Western blot analyses on lysates of BMM infected with either *L*. *donovani* or *L*. *major* promastigotes showed that the 63 kDa form of GP63 was processed into a ~42 kDa catalytically inactive form whereas the levels of LPG decreased gradually over time (Fig 1C), consistent with the down-modulation of these molecules during the differentiation of promastigotes into the mammalian stage amastigotes [20, 21]. Using *L*. *donovani* LPG-defective (Δ*lpg1*) metacyclic promastigotes, which retain the ability to express the less abundant surface proteophosphoglycan, we observed that this PG also traffics out of the PV and that absence of LPG had no impact on the spread of GP63 within infected cells (S1B Fig). Additionally, infection of BMM with *L*. *major* metacyclic promastigotes expressing a catalytically inactive GP63 (Δ*gp63*+*gp63*^*E265A*^) indicated that the catalytic activity of GP63 is not required for its dispersal and that of LPG beyond the PV (S2 Fig). Consistent with previous reports [18], we observed that purified LPG coated on zymosan trafficked out the PV (S1C Fig), indicating that distribution of the *Leishmania* surface coat beyond the PV is not a parasite-driven process.

### Shedding and delivery of components of the promastigote surface coat inside macrophages require phagocytosis

**Fig 2:**
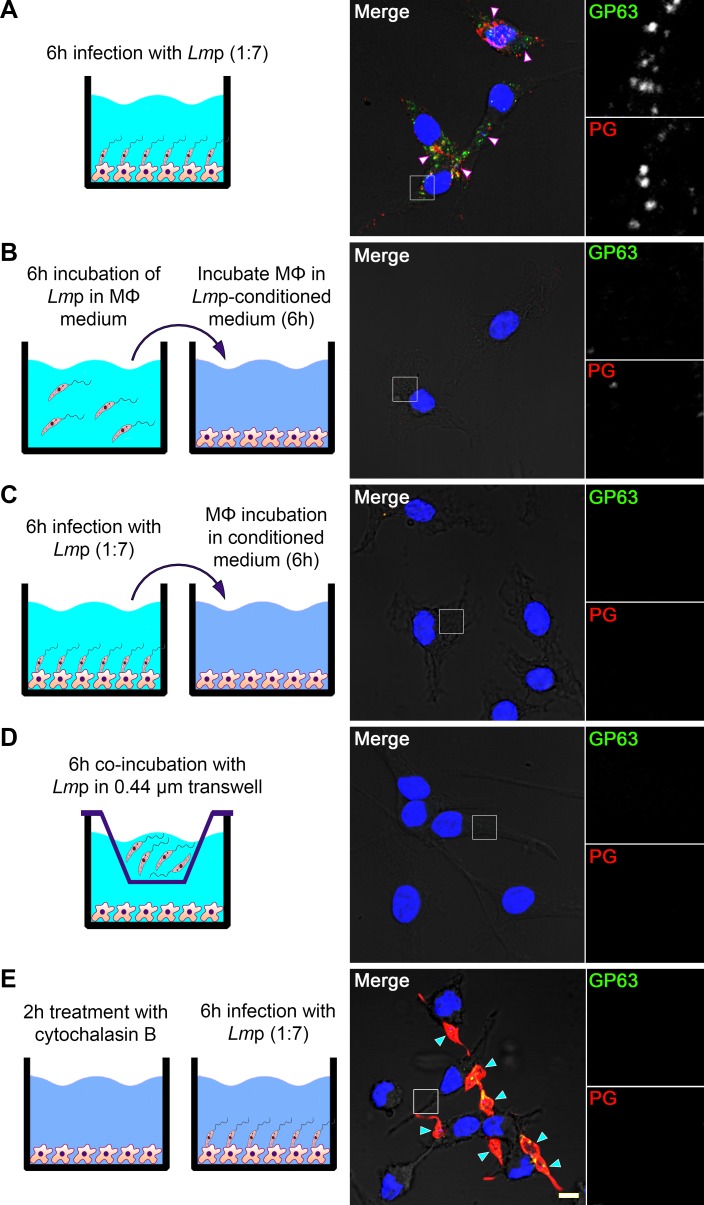
Trafficking of GP63 and PGs within macrophages requires parasite internalization. BMM were either infected with opsonized *L*. *major* Δ*gp63*+*gp63* metacyclic promastigotes **(A)**, incubated with conditioned medium from promastigotes cultured at 37°C **(B)**, or from infected macrophages **(C)**, for 6 h. Contact dependence was tested by incubating BMM with promastigotes separated by a 0.44 μm transwell **(D)**, and the requirement for entry was tested by pre-incubating BMM with cytochalasin B to inhibit phagocytosis **(E)**. Redistribution of GP63 (green) and PGs (red) in the cytoplasm of infected cells was observed via immunofluorescence; DNA is in blue. 5X-enlarged channel-split insets of representative cytoplasmic regions are shown. White and cyan arrowheads denote internalized and non-internalized parasites, respectively. Bar, 5 μm. These results are representative of two independent experiments.

**Fig 3:**
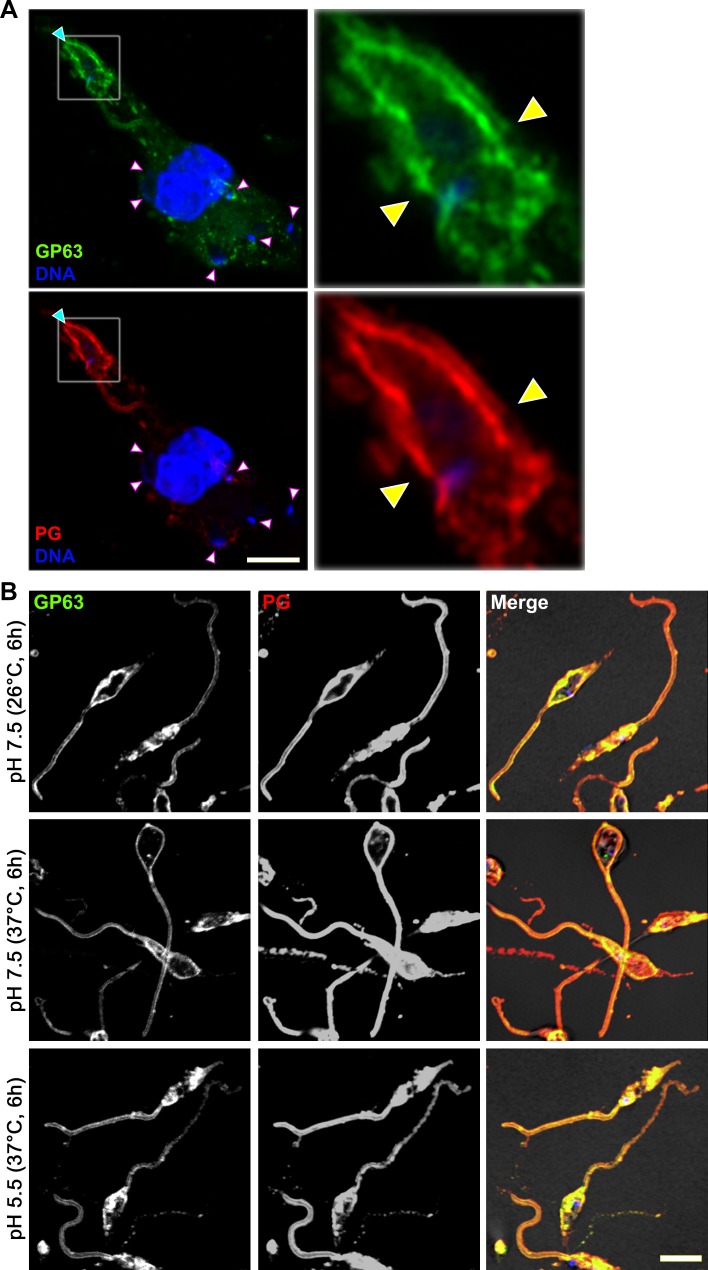
The phagosomal environment triggers shedding of GP63 and PG. **(A)** BMM were infected with opsonized *L*. *major* metacyclic promastigotes and enhanced resolution imaging was used to image the distribution of GP63 (green) and PGs (red) on internalized (white arrowheads) and semi-internalized (cyan arrowhead) promastigotes. A 4.25X-enlarged inset of the semi-internalized promastigote is shown, with the locations of the phagocytic cup denoted by yellow arrowheads. **(B)** *L*. *major* metacyclic promastigotes were incubated in complete medium at pH 7.5 or 5.5 for 6 h at either 37°C or 26°C. Localization of GP63 (green) and PG (red) was examined by immunofluorescence confocal microscopy. These results are representative of two independent experiments. DNA is in blue; bar, 5 μm.

To access host cell locations, virulence factors produced by vacuolar pathogens must cross the PV membrane. Bacterial pathogens have evolved complex nanomachines to inject effector proteins into the cytosol of eukaryotic cells [26], whereas apicomplexan parasites deliver their virulence factors through rhoptries [25]. In *Leishmania*, no such specialized secretion system has been described. Instead, an exosome-based secretion system was proposed as a general mechanism for protein secretion [31]. The finding that GP63 and LPG are constituents of *Leishmania* exosomes [31–33] prompted us to explore the potential role of these extracellular vesicles in the transfer of GP63 and PGs within host cells. As shown in Fig 2, we did not detect notable levels of these molecules in BMM incubated with conditioned medium from promastigotes cultured at 37°C (enriched in exosomes) or from infected macrophages, or when contact between *Leishmania* and BMM was prevented by a 0.44 μm transwell. These observations indicate that extracellular vesicles released by non-internalized promastigotes are not significantly contributing to the delivery of GP63 and PGs to the cytosol of macrophages. Equally, GP63 and PGs largely remained at the parasite surface when phagocytosis was inhibited by cytochalasin B (Fig 2E), indicating that shedding and redistribution of these molecules within host cells depends on parasite internalization. To address this issue, we used enhanced resolution imaging to visualize GP63 and PGs on the surface of metacyclic *L*. *major* promastigotes during phagocytosis by BMM. We observed that the uniform PGs and GP63 staining on metacyclic promastigotes became punctate once the parasites were phagocytosed, due to shedding from the parasite surface and transport beyond the PV (Fig 3A). These results indicate that environmental conditions present in the PV trigger the shedding of components of the parasite surface coat prior to their spreading out of the PV. Shift in temperature (from 26°C to 37°C) and drop in pH (from pH 7.0 to pH 5.5) are considered the main signals that induce the differentiation of promastigotes into amastigotes [34] and were shown to increase the release of exosomes from promastigotes [31, 35]. We therefore assessed whether these conditions were sufficient to trigger the shedding of GP63 and PGs from the surface of *L*. *major* metacyclic promastigotes. As shown on Fig 3B, increased temperature and drop in pH had no effect on the surface distribution of GP63 and PGs. Collectively, these results indicate that shedding of the promastigote surface coat and the subsequent transfer of GP63 and PGs inside the host cell is triggered by phagosomal cues that remain to be identified.

### GP63 and LPG localize within the endoplasmic reticulum of infected macrophages

**Fig 4:**
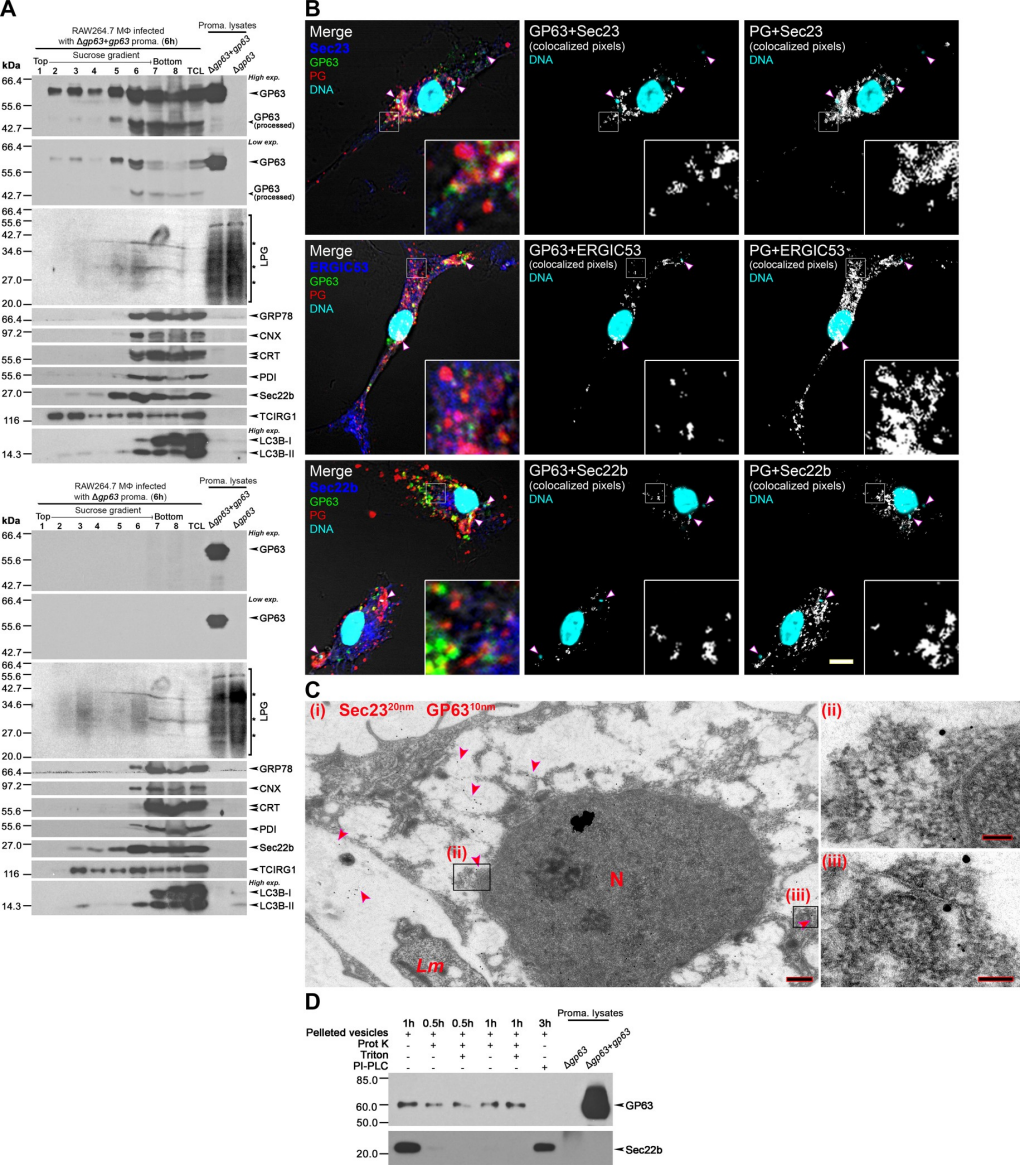
GP63 and PGs are present in vesicles that co-occur with ER and ERGIC markers. RAW264.7 macrophages were infected with either *L*. *major* Δ*gp63*+*gp63* or Δ*gp63* opsonized metacyclic promastigotes for 6 h. Lysates were placed in a sucrose gradient and fractionated from the top. **(A)** Western blot showing the presence of GP63 and LPG in light (2–4) or denser fractions (5–8). GRP78, CNX, CRT, and PDI were used as ER markers, Sec22b as an ERGIC marker, and TCIRG1 as a maker of endosomes and lysosomes. Light vesicle-containing fractions are delimited by the exclusive appearance of LC3B-II, which is membrane-bound. TCL, total cell lysate. **(B)** BMM were infected with opsonized *L*. *major* Δ*gp63*+*gp63* metacyclic promastigotes for 6 h and the colocalization (white pixels, middle and rightmost panels) of GP63 (green) or PGs (red) with ER marker Sec23 (blue), or ERGIC markers ERGIC53 (blue) and Sec22b (blue) was assessed by confocal immunofluorescence microscopy. DNA is in cyan. 5X-enlarged insets of representative cytoplasmic regions are shown. White arrowheads denote internalized parasites. Bar, 5 μm. **(C)** (i) Immuno-electron microscopy image (bar, 500 nm) of a representative 6 h-infected BMM stained for Sec23 (20 nm nanoparticles) and GP63 (10 nm nanoparticles). Red arrowheads denote regions where both Sec23 and GP63 were in close proximity, two of which were magnified (bar, 100 nm) in rightmost panels (ii) and (iii). *Lm*, *L*. *major*-containing PV; N, BMM nucleus. **(D)** Enzyme protection assay with vesicles that were pelleted from fraction 6 and treated with Prot K ± Triton X-100 or PI-PLC. The protection of GP63 from these enzymes was compared to that of the host’s Sec22b, which faces the cytoplasmic side and is not GPI-anchored. These results are representative of at least two independent experiments.

Previous studies revealed that both LPG and GP63 associate with lipid microdomains of host cell membranes to impair host cell processes [8, 16]. Apart from the reported presence of GP63 in the perinuclear area of infected macrophages [8], little is known with respect to the sub-cellular localization of PGs and GP63 within host cells. To address this issue, we resolved lysates of RAW264.7 macrophages infected with *L*. *major* Δ*gp63*+*gp63* or Δ*gp63* metacyclic promastigotes on discontinuous sucrose gradients and assessed their distribution by Western blot analysis. Since these fractionation experiments require a large number of macrophages, we used the macrophage cell line RAW264.7 in lieu of BMM. GP63 and LPG were present in fractions 2 to 8, with fractions 5 and 6 showing the strongest signal and Δ*gp63*-infected cells exhibiting higher LPG levels (Fig 4A and S3B and S3C Fig). This finding indicated that both molecules sedimented with light-density vesicles (S3A Fig), consistent with the presence of the membrane-bound protein LC3B-II [36]. GP63 and LPG were also present in denser fractions containing endoplasmic reticulum (ER) proteins BiP (GRP78), calreticulin (CRT), calnexin (CNX), protein disulfide isomerase (PDI), and the ER-Golgi intermediate compartment (ERGIC) soluble *N*-ethylmaleimide-sensitive-factor attachment protein receptor (SNARE) Sec22b. Confocal microscopy analyses revealed that GP63 and PGs colocalize with ERGIC (ERGIC53, Sec22b), and ER (CRT, PDI, Sec23) proteins in infected BMM (Fig 4B and S4A Fig). Moreover, immunogold electron microscopy revealed that GP63 and Sec23 co-occur in tubulovesicular structures in infected cells (Fig 4C and S11B Fig). Colocalization of GP63 and PGs with GM1-enriched lipid rafts was also observed, as previously reported [3, 16]. Of note, none of the ER/ERGIC proteins analyzed were targeted by GP63 (S4B Fig). To determine whether GP63 was oriented into the lumen of those vesicles or exposed to the cytoplasm, we incubated pelleted vesicles with either proteinase K (Prot K) or phospholipase C (PI-PLC) before assessing the presence of GP63 by Western blot analysis (Fig 4D). Although GP63 was resistant to Prot K digestion even in the presence of Triton X-100, it was released from the vesicles by PI-PLC, due to the cleavage of its GPI anchor. In contrast, the cytosol-facing integral membrane protein SNARE Sec22b [37] was only sensitive to Prot K treatment. The processed form of GP63 (~42kDa) was found almost exclusively on fractions 6–8 (which contain large organelles and protein deposits). Since we did not detect that fragment in pelleted membrane (129536.6 *g*) from fraction 6, processed GP63 may not be membrane-anchored. These results indicate that following their release from the parasite surface, GP63 and LPG are exported from the PV to the ER/ERGIC. In addition, GP63 is present on the cytoplasmic face of the vesicles, consistent with the ability of this protease to cleave host cell proteins.

### Exit of GP63 and LPG from the PV requires ER-Golgi trafficking and the SNAREs Sec22b and syntaxin-5 (Stx5)

**Fig 5:**
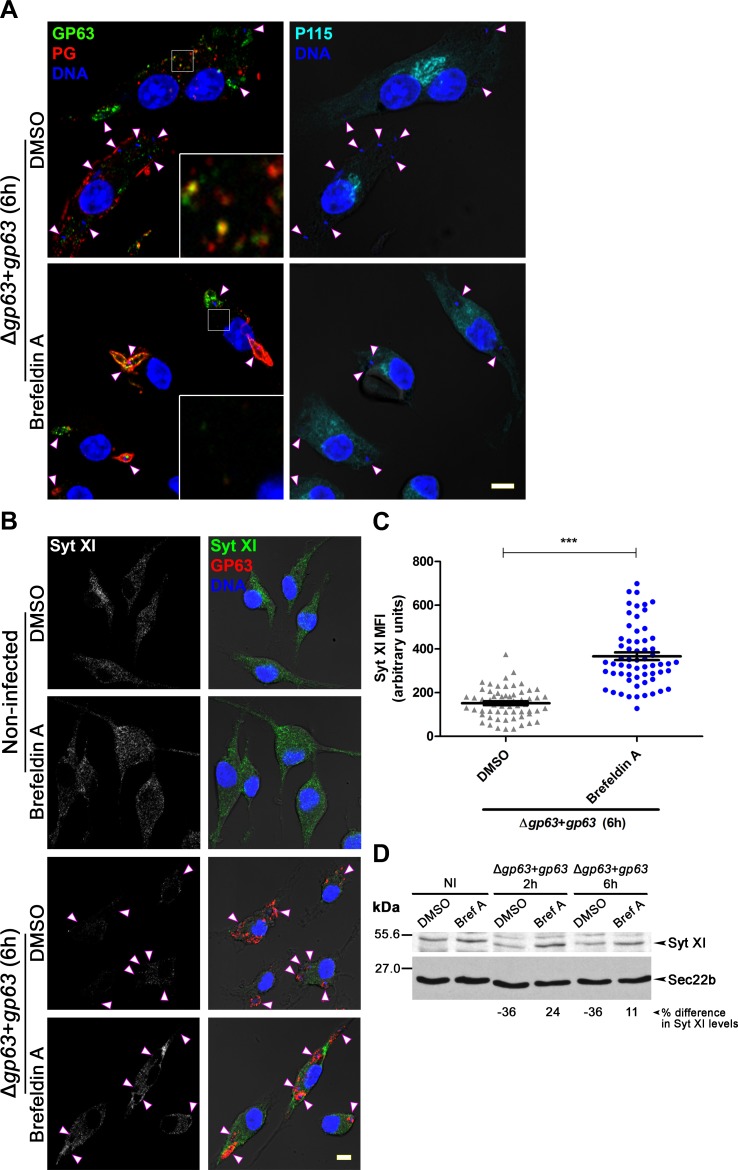
Pharmacological inhibition of ER-Golgi trafficking hampers the redistribution of GP63 and PGs and the cleavage of Syt XI. **(A)** BMM were treated with brefeldin A or DMSO prior to infection with opsonized *L*. *major* Δ*gp63*+*gp63* metacyclic promastigotes for 6 h. GP63 is shown in green, PGs in red and P115 (a reporter of ER-Golgi disruption) in cyan. 5X-enlarged insets of representative cytoplasmic regions are shown. **(B)** Immunofluorescence showing the impact of brefeldin A or DMSO treatment on the degradation of Syt XI (green); GP63 is shown in red. In panels (A) and (B), white arrowheads denote internalized parasites and DNA is in blue. Bar, 5 μm. **(C)** Quantification of Syt XI levels in infected brefeldin A- or DMSO-treated cells. Data are presented as mean ± s.e.m. of n = 4 experiments (≥15 cells per experiment), with each point representing the MFI of a single cell. ***, *p* < 0.001; MFI, mean fluorescence intensity. **(D)** Lysates from brefeldin A- and DMSO-treated infected cells were examined by Western blot to evaluate the cleavage of Syt XI. The % difference in Syt XI levels represents the % difference in band intensities of infected vs. non-infected (NI) macrophages. Band intensities were normalized to Sec22b levels and a negative value is indicative of cleavage. Results are representative of at least two independent experiments. Bref A, brefeldin A.

The ER closely interacts with phagosomes and mediates the export of intraphagosomal proteins [38]. To investigate the potential role of the ER and the ERGIC in the trafficking of GP63 and PGs beyond the PV, we employed the inhibitor of ER-Golgi trafficking brefeldin A [39, 40]. Disruption of Golgi by brefeldin A was verified by the dispersal of *cis*-Golgi marker P115 [36] (Fig 5A). Following infection of brefeldin A-pretreated macrophages with metacyclic *L*. *major* Δ*gp63*+*gp63* promastigotes, trafficking of GP63 and PGs out of the PV was abrogated (Fig 5A). Similarly, brefeldin A pretreatment inhibited the trafficking of LPG from LPG-coated zymosan out of the phagosome (S7 Fig). Consistent with the notion that GP63 must exit the PV to cleave host cell molecules, cleavage of the macrophage membrane fusion regulator Synaptotagmin XI (Syt XI) (Fig 5B and 5C) and VAMP3 & VAMP8 (S5 Fig) by GP63 [6, 7] was impaired in brefeldin A-treated macrophages (Fig 5B and 5C). These results indicate that GP63 and PGs spread beyond the PV through a mechanism involving vesicular trafficking between the PV and the ER/ERGIC.

**Fig 6:**
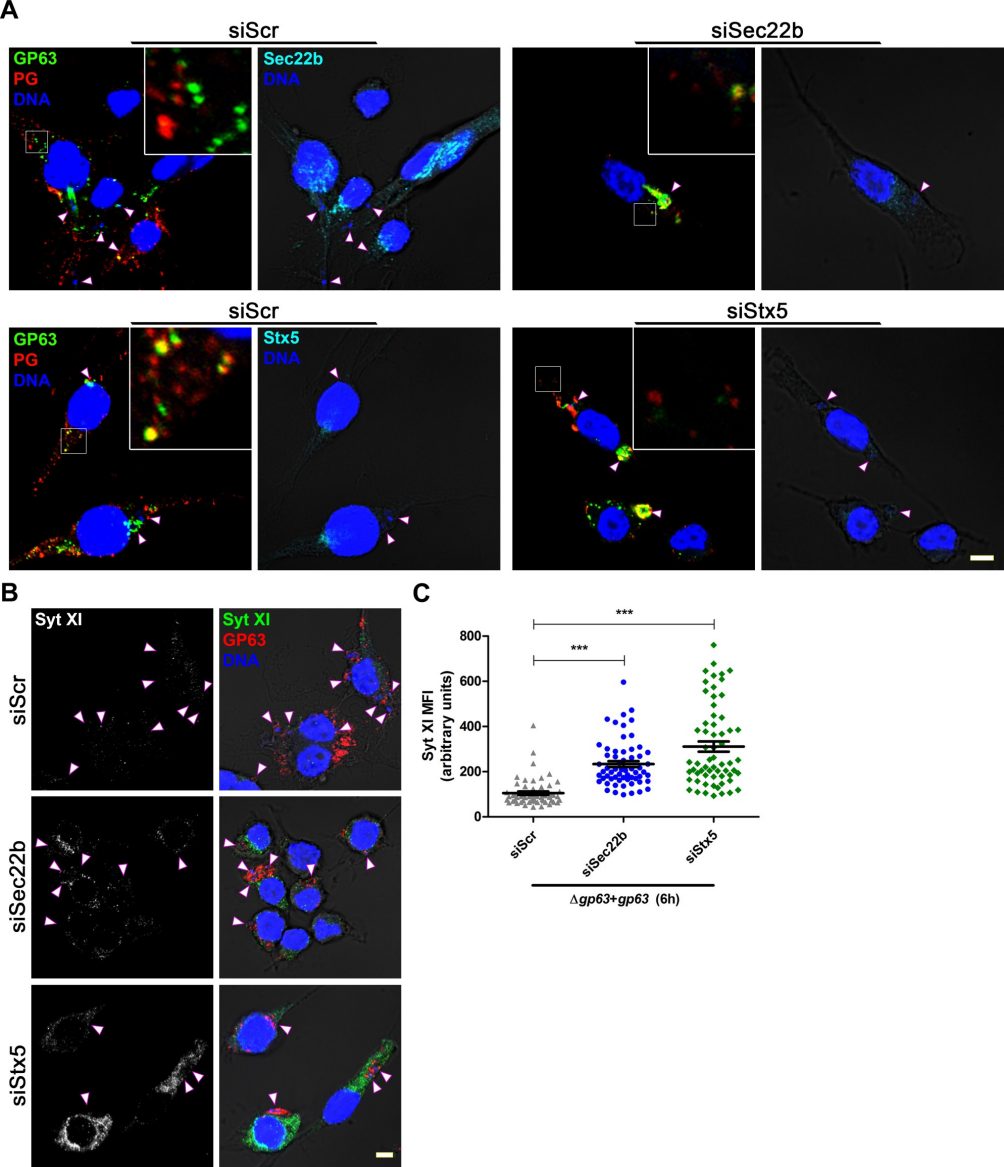
Knockdown of Sec22b and Stx5 abrogates the redistribution of GP63 and PGs. **(A)** RAW264.7 macrophages transfected with siRNAs targeting Sec22b (cyan), Stx5 (cyan) or a scrambled sequence (Scr) were infected with opsonized *L*. *major* Δ*gp63*+*gp63* metacyclic promastigotes for 6 h. The effect of these knockdowns on the redistribution of GP63 (green) and PGs (red) was visualized. 5X-enlarged insets of representative cytoplasmic regions are shown. **(B)** Immunofluorescence showing the impact of Sec22b- or Stx5-knockdown (KD) on the degradation of Syt XI (green); GP63 is shown in red. In panels (A) and (B), white arrowheads denote internalized parasites and DNA is in blue. Bar, 5 μm. **(C)** Quantification of Syt XI levels in infected Sec22b- or Stx5-KD cells. Data are presented as mean ± s.e.m. of n = 4 experiments (≥15 cells per experiment), with each point representing the MFI of a single cell. ***, *p* < 0.001; MFI, mean fluorescence intensity.

The ER/ERGIC-resident SNAREs Sec22b and Stx5 play a key role in phagosome maturation and function by regulating the delivery of ER and ERGIC resident proteins to phagosomes [41, 42]. To test whether Sec22b and Stx5 also operate by regulating transport of phagosome cargo to the ER/ERGIC, we infected the mouse macrophage cell line RAW 264.7 treated with siRNAs to Sec22b or Stx5 with *L*. *major* Δ*gp63*+*gp63* metacyclic promastigotes for 6 h. As shown in Fig 6A–6D, exit of GP63 and PGs out of the PV and cleavage of Syt XI were impaired in cells expressing reduced levels of either SNARE. Similar to brefeldin A, knockdown of Sec22b also impaired the trafficking of LPG from LPG-coated zymosan beyond the PV (S6 Fig). To further confirm the role of Sec22b in the redistribution of *Leishmania* virulence factors beyond the PV, we infected the dendritic cell line JAWS-II transduced with short hairpin RNAs (shRNAs) to knock down Sec22b [41]. Cells transduced with a scrambled shRNA were used as a control. As shown in S7A–S7C Fig, redistribution of GP63 and PGs and cleavage of GP63 substrates were impaired in the absence of Sec22b. GP63 redistribution within infected cells was also assessed by Western blot analysis on sucrose gradient fractions. Absence of Sec22b prevented GP63 and LPG to sediment in lighter fractions, as was the case in control JAWS-II cells (S8 Fig). Together, these data indicate that the ER/ERGIC-resident SNAREs Sec22b and its cognate SNARE Stx5 are part of the host cell machinery that mediates the redistribution of *Leishmania* virulence factors beyond the PV.

## Discussion

To manipulate host cell processes and pathways, intracellular pathogens translocate effector proteins in the cytosol of infected cells through the action of specialized secretion systems [25, 26]. The fact that *Leishmania* has no known such dedicated secretion apparatus prompted us to investigate the strategy used by these parasites to transfer virulence factors from their surface coat into their host cells. In the present study, we report that *Leishmania* promastigotes take advantage of the trafficking pathway between the PV and the ER/ERGIC to deliver LPG and GP63 beyond the PV where they can alter host cell processes (S9 Fig).

The surface coat of *Leishmania* promastigotes plays an essential role in the ability of this parasite to colonize phagocytic cells [5, 11]. To exert their action on host cell pathways, LPG and GP63 must first be shed from the parasite surface before spreading across infected cells. Our results revealed that internalization by phagocytic cells is essential for the release of both LPG and GP63 from the surface of metacyclic promastigotes and their subsequent trafficking within infected cells. Indeed, we did not detect GP63 or LPG in macrophages incubated with metacyclic promastigote-conditioned medium or when phagocytosis was pharmacologically inhibited. These observations suggest a mechanism by which metacyclic promastigotes shed their surface coat in response to cues provided by the phagosomal environment. The finding that *Leishmania* promastigotes secrete exosomes containing GP63 and LPG [27, 28] raised the possibility that these molecules are transferred to host cells through exosomes [28]. Although attractive, this model does not take into consideration the rapid shedding of the metacyclic promastigote surface coat that occurs upon internalization by macrophages. Indeed, exosomes have an intracellular origin and their release is slow [43]. By contrast, the possibility that LPG and GP63 are shed through the formation of ectosomes induced by signals provided by the PV environment is consistent with the rapid disappearance of these two surface coat molecules. Shedding of ectosomes occurs rapidly and consists in the removal of portions of the plasma membrane. Interestingly, high rate of ectosome shedding is associated to reduced cell volume [43], a feature of the promastigote-to-amastigote differentiation process. Thus, one can envision that shedding of the promastigote surface coat inside the PV is one of the early events associated to the differentiation of metacyclic promastigotes into amastigote forms (in which LPG and GP63 are absent or down-modulated) [20, 21]. It is generally accepted that temperature change, which occurs when the parasite is transferred from the vector to the mammalian host, and drop in phagosomal pH constitute important cues to trigger the differentiation of promastigotes into amastigotes [34]. Our finding showing that incubation of *L*. *major* promastigotes at 37°C and pH 5.5 for 6 h had no noticeable effect on the integrity of the surface coat indicates that phagosomal cues that trigger shedding of the surface coat remain to be identified.

As LPG and GP63 are shed from the surface of internalized metacyclic promastigotes, they spread beyond the PV. Through cell fractionation and immuno-colocalization studies, we observed that a significant portion of GP63 and LPG released by the parasites traffics to the ER and the ERGIC of infected cells. The fact that phagosomes interact with the ER and the ERGIC during and after phagocytosis [41, 44, 45] provides a mechanism for the transfer of GP63 and LPG from the PV to these organelles. Indeed, disruption of the traffic between the ER and the Golgi with brefeldin A abrogated the exit of GP63 and LPG from the PV, suggesting that vesicular trafficking between the PV and the ER/ERGIC plays a key role in this process. This observation may provide a mechanistic explanation for the previously reported localization of GP63 at the nuclear envelope of infected cells, since this structure is continuous to the ER [46]. The ER/ERGIC SNARE Sec22b, which regulates ER-to-Golgi traffic, also controls the recruitment of ER and ERGIC resident proteins to phagosomes [41]. Our results indicate that Sec22b and its partner Stx5 are key components of the molecular machinery that mediates the transport of cargo from PV to the ER/ERGIC in the case of GP63 and LPG. In addition to mediating trafficking of LPG and GP63 beyond the PV, this transport pathway is essential for the cleavage of GP63 targets, illustrating its importance for the pathogenesis of *Leishmania*. In this regard, the demonstration that GP63 faces the host cell cytoplasm is consistent with its ability to cleave host cell substrates, but the underlying mechanism remains elusive. One possible model is that following its release as part of extracellular vesicles from the promastigote surface, GP63 is already present on the outside face of these vesicles. Through an unknown mechanism involving the secretory pathway, these vesicles harboring GP63 are transferred from the PV to the ER/ERGIC, and possibly into other host cell compartments. Future studies will be necessary to elucidate this issue. Through the action of specialized effectors, bacterial pathogens such as *Legionella* actively subvert Sec22b-dependent vesicular transport pathway that recruits ER-derived membranes to the phagosome [47, 48]. In the case of *Leishmania*, the observation that LPG coated on inert particles such as zymosan redistributes within macrophages [18] strongly suggests that Sec22b-dependent dispersal of the *Leishmania* surface coat beyond the PV is not a parasite-driven process. Of interest, it was recently reported that LPG triggers caspase-11 activation in the macrophage cytoplasm through an unknown mechanism [49]. Whether the Sec22b-dependent vesicular trafficking between the PV and the ER allows LPG to access the macrophage cytosol and engage caspase-11 activation is a possibility that deserves consideration. Future studies will be necessary to further clarify these issues.

In eukaryotic cells, the ER represents the largest organelle and interacts with virtually all other organelles through membrane contact sites, which are involved in the transport of ions, lipids, and proteins [50, 51]. For a vacuolar pathogen, the ER may thus represent a highly efficient gateway through which virulence factors may further spread to host cell organelles to subvert microbicidal and immune functions.

## Materials and methods

### Ethics statement

Animal work was performed as stipulated by protocols 1706–06 and 1706–07, which were approved by the *Comité Institutionel de Protection des Animaux* of the INRS-Institut Armand-Frappier. These protocols respect procedures on animal practice promulgated by the Canadian Council on Animal Care, described in the Guide to the Care and Use of Experimental Animals.

### Antibodies, plasmids and inhibitors

Rabbit polyclonal antibodies anti-Sec22b, -Stx5, -VAMP3 and -VAMP8 were obtained from Synaptic Systems; anti-β-actin, -ERGIC53 and -microtubule-associated protein 1 light chain 3 (LC3B) from Sigma; anti-PDI and -CNX from Enzo Life Sciences; anti-CRT from ThermoFisher; anti-glucose-regulated protein 78 (GRP78/BiP) from BD Signalling; and anti-Sec23 and T-cell immune regulator 1 (TCIRG1) from Abcam. The rabbit anti-Syt XI polyclonal antibody was previously described [52]. The mouse anti-phosphoglycan (Galβ1,4Manα1-PO_4_) CA7AE monoclonal antibody [53] was from Cedarlane. The mouse anti-GP63 monoclonal antibodies #96 (IgG2A, used in confocal microscopy) and #235 (used in Western blotting) [54, 55], and the pLeishNeoGP63.1^E265A^ construct (which expresses catalytically-inactive GP63) [56] were kindly provided by Dr. W. Robert McMaster (University of British Columbia). Pharmacological inhibitors brefeldin A (35 μg/ml) (Molecular Probes) and cytochalasin B (5 μM) (Sigma) were reconstituted in DMSO (Bioshop).

### Mammalian cell culture

Bone marrow-derived macrophages (BMM), dendritic cells (BMDC) and inflammatory monocytes were differentiated from the bone marrow of 6- to 8-week old female C57BL/6 mice. BMM were differentiated during 7 days in complete DMEM (containing L-glutamine (Life Technologies), 10% v/v heat-inactivated foetal bovine serum (FBS) (Life Technologies), 10 mM HEPES (Bioshop) at pH 7.4, and penicillin-streptomycin (Life Technologies)) supplemented with 15% v/v L929 cell-conditioned medium (LCM) as a source of macrophage colony-stimulating factor. To render BMM quiescent prior to experiments, cells were transferred to tissue culture-treated plates and kept for 16 h in complete DMEM without LCM [57]. BMDCs were differentiated during 7 days in RPMI (Life technologies) containing 10% heat-inactivated FBS, 10 mM HEPES at pH 7.4, antibiotics and 10% v/v X63 cell-conditioned medium as a source of granulate-macrophage colony-stimulating factor [58] [59]. Inflammatory monocytes were differentiated using complete DMEM containing 15% LCM for three days. Four days post-differentiation, monocytes were washed and incubated for 3 h with 100 U/ml IFN-γ prior to use [60]. The mouse macrophage cell line RAW264.7 (obtained from the American Type Culture Collection), clone D3 was cultured in complete DMEM. JAWS-II dendritic cell-like lines stably transduced with shRNA for Sec22b or with shScr (control) [41] were a kind gift from Dr. Sebastian Amigorena (Institut Curie). JAWS-II cells were cultured in RPMI containing 20% heat-inactivated FBS, 10 mM HEPES at pH 7.4, 10% X63 cell-conditioned medium and 40 μg/ml puromycin (Bioshop). All mammalian cells were kept in a humidified 37°C incubator with 5% CO_2_.

### Parasite culture

The *L*. *major* strains used in this study were passaged in mice to maintain their virulence. Amastigotes recovered from ear dermis lesions of infected BALB/c mice were differentiated into promastigotes in *Leishmania* medium [M199-1X (Sigma) with 10% heat-inactivated FBS, 40 mM HEPES at pH 7.4, 100 μM hypoxanthine, 5 μM hemin, 3 μM biopterin, 1 μM biotin, and antibiotics] in a 26°C incubator. *L*. *major* Seidman (MHOM/SN/74) NIH clone A2 (A2WF) promastigotes (WT, Δ*gp63*, and Δ*gp63*+*gp63*) were kindly provided by Dr. W. Robert McMaster (University of British Columbia). The pLeishNeoGP63.1^E265A^ construct was electroporated into Δ*gp63* promastigotes [61], selected with G418, and clones expressing similar GP63 levels to Δ*gp63*+*gp63* promastigotes were used for experiments. The Δ*gp63*+*gp63* and Δ*gp63*+*gp63*^*E265A*^ *L*. *major* promastigotes were grown in *Leishmania* medium supplemented with 100 μg/ml G418 (Life Technologies). *L*. *donovani* LV9 (MHOM/ET/67/Hu3:LV9, obtained from Dr. Greg Matlashewski, McGill University) amastigotes were isolated from the spleens of infected HsdHan:AURA hamsters (Harlan Sprague Dawley Inc.) and were differentiated into promastigotes in *Leishmania* medium in a 26°C incubator. WT and isogenic LPG-defective Δ*lpg1 L*. *donovani* LV9 promastigotes [62] were grown in *Leishmania* medium. *L*. *braziliensis* M15991 (MHOM/BR/1996/M15991) and *L*. *infantum* BA262 (MCAN/BR/89/BA262) were kindly provided by Dr. Rodrigo P. Soares (Centro de Pesquisas René Rachou/Fundação Oswaldo Cruz) and Dr. Valeria M. Borges (Centro de Pesquisas Gonçalo Moniz/Fundação Oswaldo Cruz), respectively. These New World strains were also grown in *Leishmania* medium.

### siRNA knockdown

RAW264.7 macrophages in the second passage were reverse-transfected with Lipofectamine RNAiMAX (Thermo Fisher Scientific) in 24-well plates, as per the manufacturer’s protocol. Cells were transfected with non-targeting siRNA, siRNA to Sec22b (Thermo Fisher Scientific) or Stx5 (GE Healthcare Dharmacon). Cells were incubated with lipofectamine and siRNAs at a final concentration of 25 nM in a 600 μl final volume of complete DMEM with no antibiotics for 72 h. Then, cells were washed with complete medium and infected. BLAST searches were done to confirm that the siRNAs employed targeted only the mRNAs of interest.

### Infections and pH-temperature incubations

Metacyclic promastigotes were enriched from cultures of freshly differentiated promastigotes in late stationary phase [63]. Briefly, a 2 ml cushion of 40% w/v Ficoll PM400 (GE Healthcare) was deposited on a 15 ml tube, followed by a 2 ml layer of 10% Ficoll PM400 in M199-1X. Late stationary phase promastigotes were resuspended in a 2 ml volume of DMEM with no serum, and overlaid onto the 10% Ficoll layer. After a 10 min spin, metacyclic promastigotes were collected from the DMEM-10% Ficoll interphase, which regularly contained 10% of the input population. These metacyclic promastigotes displayed an overall higher GP63 activity and longer LPG molecules than log phase parasites and were infective to, and survived in BMM (S10 Fig). Metacyclic promastigotes were opsonised with C5-deficient serum from DBA/2, resuspended in cold complete DMEM and fed to macrophages or JAWS-II cells at 4°C for 5 min [64], followed by a 2 min spin at 298.2 *g* in a Sorvall RT7 centrifuge. Parasite internalization was triggered by transferring cells to 37°C [7, 65]. After 2 h, non-internalized parasites were washed 3X with warm medium. Cells were then washed and prepared for lysis or confocal immunofluorescence microscopy. Parasite survival was assessed by scoring intracellular parasites in eosin-hematoxylin-stained BMM. Alternatively, macrophages were fed with LPG-coated zymosan (Sigma) as previously described [7, 18, 66]. In transwell experiments, metacyclic promastigotes and macrophages were separated by a cell culture insert (Fischer Scientific) containing a membrane with 0.44 μm pores. To test the effect of pH and temperature on the shedding of GP63 and PG, metacyclic promastigotes were spun onto poly-L-lysine coverslips (BD) and incubated for 6 h at 26°C or 37°C in complete medium at pH 7.5 or 5.5 [34]. Parasites were then washed and prepared for immunofluorescence microscopy.

Air pouch exudates containing infected neutrophils were obtained from 6 to 8-week old mice. Briefly, 3 ml of sterile air was injected subcutaneously, at days 0 and 3, in the backs of anesthetized mice [67]. At day 6, 50x10^6^ *L*. *major* Δ*gp63*+*gp63* promastigotes in 0.5 ml HBSS were injected in the air pouch. Six hours post-injection, mice were sacrificed and exudates were obtained by washing the air pouches with HBSS-EDTA. Exudate cells were washed with complete medium, spun onto poly-L-lysine coverslips, and incubated at 37°C for 20 min. Coverslips were then washed and prepared for microscopy.

### Electrophoresis, Western blotting and zymography

Prior to lysis, adherent macrophages or parasites were placed on ice and washed with PBS containing 1 mM sodium orthovanadate and 5 mM 1,10-phenanthroline (Sigma). Macrophages were scraped in the presence of lysis buffer containing 1% NP-40, 50 mM Tris-HCl (pH 7.5), 150 mM NaCl, 1 mM EDTA (pH 8), 10 mM 1,10-phenanthroline, and phosphatase and protease inhibitors (Roche). In the case of the semi-adherent JAWS-II cells, they were spun and pelleted 3X at 101.5 *g* in 5 ml of cold PBS (containing orthovanadate and phenanthroline) prior to lysis. After incubation at -70°C, lysates were centrifuged for 15 min to remove insoluble matter. After protein quantification, 30 μg of protein was boiled (100°C) for 6 min in SDS sample buffer and migrated in SDS-PAGE gels. Proteins were transferred onto Hybond-ECL membranes (Amersham Biosciences), blocked for 2 h in TBS1X-0.1% Tween containing 5% BSA, incubated with primary antibodies (diluted in TBS1X-0.1% Tween containing 5% BSA) overnight at 4°C, and thence with suitable HRP-conjugated secondary antibodies for 1h at room temperature. Membranes were incubated in ECL (GE Healthcare) and immunodetection was achieved via chemiluminescence [7]. Densitometric analysis of Western blot bands was done using the AlphaEase FC software (Alpha Innotech) and heat maps were generated using R [36]. GP63 activity was assayed via gelatin zymography [68]. Briefly, 10 μg of infected cell lysate, or 2 μg of promastigote lysate were incubated at 50°C for 5 min in sample buffer without DTT and then migrated in 10% SDS-PAGE gels containing 0.12% gelatin (Sigma). Gels were incubated for 2 h in the presence of 50 mM Tris pH 7.4, 2.5% Triton X-100, 5 mM CaCl_2_ and 1 mM ZnCl_2_, followed by an overnight incubation at 37°C in a buffer containing 50 mM Tris pH 7.4, 5 mM CaCl_2_, 1 mM ZnCl_2_ and 0.01% NaN_3_. Protease activity was visualized by staining the gels with 0.5% Coomassie (Sigma).

### Sucrose gradient flotation assays

Infected macrophages or JAWS-II cells (50x10^6^) were washed and resuspended in a hypotonic solution consisting of 20 mM Tris at pH 7.33, 10 mM 1,10-phenanthroline and 1.5X protease inhibitors and incubated on ice for 30 min. Mechanical lysis was effectuated by passing this suspension 12X through a 1 ml syringe mounted with a 27G needle [69]. To this homogenate, NaCl was added to a final concentration of 0.1 M. After a 15 min centrifugation at 5000 *g* and 4°C, the postnuclear supernatant was passed through a 0.22 μm filter to eliminate unwashed parasites and phagosomes. For the flotation assay (discontinuous sucrose gradients), equal quantities (~3.4 mg) of protein were used for all conditions. The gradient was set up with 57% sucrose at the bottom (450 μl of lysate mixed with 1.1 ml of 80% sucrose), followed by 35% sucrose and then TBS at the top of the tube. The sucrose solutions contained 1.5X protease inhibitors and 1,10-phenanthroline at 10 mM. Ultracentrifugation was carried out for 16 h at 4°C (95016.5 *g*) in a Beckman-Coulter Optima XL-I centrifuge using an SW 55 Ti rotor. After centrifugation, 650 μl fractions were carefully collected from the top. Fractions were then mixed with 0.1% sodium deoxycholate and precipitated with 17% TCA. Precipitated protein was washed with acetone, resuspended in 2X SDS sample buffer, boiled for 6 min at 100°C and loaded onto SDS-PAGE gels.

### Proteinase and phospholipase protection assays

Fractions from sucrose gradients were ultracentrifuged at 129536.6 *g* for 1 h at 4°C in a TLA100.3 rotor on a Beckman-Coulter table top ultracentrifuge in order to pellet vesicles. These were then carefully resuspended in filtered TBS 1X with no additives and treated with Prot K (Bioshop) at 150 μg/ml in the presence or absence of 10% Triton-X100, for 30–60 min at 4°C. Vesicles were also treated with *B*. *cereus* PI-PLC (Molecular Probes) at 5 U/ml for 3 h at 37°C. Reactions were stopped by adding protease inhibitors (1X) and PMSF (1 μM) for 5 min at 4°C, followed by sample buffer. Samples were immediately boiled at 100°C for 6 min and loaded onto SDS-PAGE gels.

### Immunofluorescence microscopy

Infected cells on coverslips were fixed with 2% paraformaldehyde (Canemco and Mirvac) for 20 min and blocked and permeabilized for 17 min with a solution of 0.1% Triton X-100, 1% BSA, 6% non-fat milk, 2% goat serum, and 50% FBS. This was followed by a 2 h incubation with primary antibodies diluted in PBS. Then, macrophages were incubated with a suitable combination of secondary antibodies (anti-rabbit AlexaFluor 647, anti-rat 568, highly cross-adsorbed anti-mouse-IgG2A 488, highly cross-adsorbed anti-mouse-IgM 568; Molecular Probes) and DAPI (Molecular Probes). G_M1_^+^ lipid rafts were stained with the cholera toxin-subunit B-AlexaFluor 647 conjugate (Molecular Probes) [18]. Coverslips were washed three times with PBS after every step. After the final wash, Fluoromount-G (Southern Biotechnology Associates) was used to mount coverslips on glass slides (Fisher), and coverslips were sealed with nail polish (Sally Hansen). Cells were imaged with the 63X objective of an LSM780 confocal microscope (Carl Zeiss Microimaging), and images were acquired in sequential scanning mode. For super resolution imaging, coverslips were imaged with an LSM880 NLO confocal microscope (Carl Zeiss Microimaging) equipped with the Airyscan module. Images were processed with the ZEN 2012 software and mounted with Adobe Photoshop. To ensure that fluorescent signals are not the product of channel cross-talk or background, matched and mismatched primary-secondary antibody incubations were performed. These incubations revealed that only appropriately-matched combinations yield the expected stainings (S11 Fig). Syt XI MFIs in infected brefeldin A- or siRNA-treated macrophages were analyzed with the Icy image analysis software [70]. MFIs were determined for regions of interest consisting of the outline of each cell, traced with the DIC channel as guide. At least 60 cells per condition were analyzed and statistical differences were evaluated using a two-tailed Student’s *t*-test (two groups) or one-way ANOVA followed by Tukey’s post-tests (three groups). Data were considered statistically significant when *p* < 0.05 and graphs were plotted with GraphPad Prism 5 (GraphPad).

### Electron microscopy

For immunogold labeling, infected or uninfected macrophages were fixed in 0.1% glutaraldehyde + 4% paraformaldehyde in a cocodylate buffer at pH 7.2, and post-fixed in 1.3% osmium tetroxide in collidine buffer. After dehydration, samples were embedded using the ERL-4221 kit (Polysciences Inc) and placed in BEEM capsules (Pelco Int). After resin polymerization, samples were cut using an ultramicrotome system (Ultratome). The thin sections were placed on nickel grids, treated with sodium metaperiodate and blocked with 1% BSA in PBS. Grids were then incubated with primary antibodies, washed, and incubated in suitable 10 nm (anti-mouse) or 20 nm (anti-rabbit) gold particle-conjugated secondary antibodies (Abcam). After washing, samples were contrasted with uranyl acetate and lead citrate and subsequently visualized using a Philips EM 300 electron microscope. Matched and mismatched antibody combinations were done to assay for the presence of non-specificity and background (S11 Fig). Electron microscopy was also used to visualize vesicular structures in sucrose gradient-fractionated lysates. A 10 μl aliquot of each fraction was spotted onto a copper grid, spun, and negatively stained using 3% phosphotungstic acid at pH 6.0. Structures were then visualized.

## Supporting information

S1 Fig The redistribution of PGs is similar to that of LPG. **(A)** To assess the trafficking and persistence of GP63 and PGs over a period of 6 to 24 h, we infected BMM with opsonized wild type *L*. *major*, *L*. *braziliensis*, and *L*. *chagasi* metacyclic promastigotes. Using immunofluorescence, the redistribution of GP63 and PGs was observed over the indicated time period. 5X-enlarged channel-split insets of representative cytoplasmic regions are shown. **(B)** To elucidate whether the trafficking of PGs differs in promastigotes that predominantly express the GPI-anchored LPG versus promastigotes that only secrete the repeating disaccharide-phosphate repeats (schema on the left). We infected BMM for 6 h with opsonized wild type and LPG-defective Δ*lpg1 L*. *donovani* metacyclic promastigotes. **(C)** To assay whether the redistribution of LPG is a parasite-dependent process, zymosan particles were coated with purified LPG and given to macrophages. Redistribution of LPG was assayed after 1 h via immunofluorescence. LPG or PGs are shown in red, GP63 in green and DNA in blue. Images are representative of two independent experiments and white arrowheads denote internalized parasites. Bar, 5 μm.
(TIF)

S2 Fig GP63 activity has no impact on the redistribution of GP63 and PGs. **(A)** To investigate whether the catalytic activity of GP63 was required for GP63 or PGs to disperse from the PV, we infected BMM with opsonized *L*. *major* metacyclic promastigotes expressing catalytically active (Δ*gp63*+*gp63*) or inactive (Δ*gp63*+*gp63*^*E265A*^) GP63. Six hours post-infection, cells were fixed and prepared for confocal microscopy. GP63 is shown in green, PGs in red and DNA in blue. White arrowheads denote internalized parasites and 5X-enlarged insets are shown. **(B)** Infected cell lysates and promastigote lysates were probed by Western blot. Murine β-actin was used for loading control. The Syt XI blot and gelatin zymography were used to evaluate GP63 activity. These results are representative of two independent experiments. DIC, differential intensity contrast image; NI, non-infected; bar, 5 μm.
(TIF)

S3 Fig GP63 and PGs cofractionate with vesicles and ER/ERGIC markers. RAW264.7 macrophages were either non-infected or infected with opsonized *L*. *major* Δ*gp63*+*gp63* metacyclic promastigotes for 2–6 h. A flotation assay was performed where cells were lysed mechanically; sucrose was overlaid over lysates and samples were ultracentrifuged for 18h. Fractions were collected from the top. **(A)** The presence of vesicles in the collected fractions from 6 h-infected cells (Δ*gp63*+*gp63*) was verified by electron microscopy and shown here; those from the other conditions were similar (not shown). Bar, 100 nm. **(B)** Western blots depicting the levels of various *Leishmania* and macrophage proteins in fractionated lysates from non-infected and 2 h-infected cells; 6 h infections are shown in Fig 4. GRP78, CNX, CRT, and PDI were used as ER markers, Sec22b as an ERGIC marker, and TCIRG1 as a maker of endosomes and lysosomes. Light vesicle-containing fractions are delimited by the exclusive appearance of LC3B-II, which is membrane-bound. The LPG band appears as a smear and asterisks (*) indicate non-specific bands of macrophage origin (see non-infected cell and promastigote lysate lanes). TCL, total cell lysate. **(C)** Densitometric analysis of flotation assay in Fig 4A and S3B Fig. To facilitate the comparison of band intensities in each condition, heat maps were produced from densitometry data. For each protein (e.g., Sec22b in non-infected cells), the band with the highest intensity was assigned a value of 1, and the other intensities in that group (fraction 1 to TCL) were normalized with respect to that band. Since there is no GP63 in non-infected cells, background from this condition was subtracted from the other conditions (infected cells). Densitometries were then normalized as above. The densitometry of the ~42 kDa fragment (GP63-processed) was also analyzed. In the case of LPG, a box encasing the smears was used to calculate the densitometries. Since there are no PGs in non-infected cells, background from this condition, including that given by the non-specific bands of macrophage origin, was subtracted from the other conditions (infected cells). TCL, total cell lysate.
(TIF)

S4 Fig GP63 and PGs colocalize with ER markers. **(A)** BMM were infected with opsonized *L*. *major* Δ*gp63*+*gp63* metacyclic promastigotes for 6h and the colocalization (white pixels, middle and rightmost panels) of GP63 (green) or PGs (red) with ER markers (blue) CRT and PDI was assessed by confocal immunofluorescence microscopy. DNA is in cyan. 5X-enlarged insets of representative cytoplasmic regions are shown. White arrowheads denote internalized parasites. Bar, 5 μm. **(B)** GP63 does not cleave resident ER and ERGIC proteins. To investigate whether ER and ERGIC proteins are cleaved by GP63, BMM were infected with opsonized *L*. *major* WT, Δ*gp63* or Δ*gp63*+*gp63* metacyclic promastigotes. The integrity of the various ER and ERGIC markers was assayed by Western blot. Results are representative of at least two independent experiments. NI, non-infected.
(TIF)

S5 Fig Pharmacological inhibition of ER-Golgi trafficking hampers the cleavage of VAMP3 and VAMP8. BMM were treated with brefeldin A or DMSO prior to infection with opsonized *L*. *major* Δ*gp63*+*gp63* metacyclic promastigotes for 6h. The impact of these treatments on the degradation of VAMP3 and VAMP8 (green) by GP63 (red) was assayed via immunofluorescence. White arrowheads denote internalized parasites and DNA is in blue. Bar, 5 μm.
(TIF)

S6 Fig Brefeldin A and Sec22b knockdown inhibit the redistribution of LPGs. To assay whether the redistribution of LPG is a host cell-dependent process, zymosan particles were coated with purified LPG and given to RAW264.7 macrophages transfected with siRNA or treated with brefeldin A. Redistribution of LPG (red) was assayed after 1 h via immunofluorescence. Sec22b is in green, DNA in blue, and the position of zymosan particles is denoted by an asterisk. Images are representative of two independent experiments; bar, 5 μm.
(TIF)

S7 Fig shRNA-mediated knockdown of Sec22b abrogates the redistribution of GP63 and PGs. **(A)** JAWS-II cells transduced with scrambled (shScr) or Sec22b shRNA (shSec22b) were infected with opsonized *L*. *major* Δ*gp63*+*gp63* metacyclic promastigotes for 6 h. The effect of Sec22 (cyan) KD on the redistribution of GP63 (green) and PGs (red) was visualized. 5X-enlarged insets of representative cytoplasmic regions are shown. **(B)** In Sec22b-KD JAWS-II cells, the degradation of Syt XI, VAMP3 and VAMP8 (green) by GP63 (red) was assayed via immunofluorescence. In panels (A) and (B), white and red arrowheads denote internalized parasites and non-infected cells, respectively. DNA is in blue; bar, 5 μm. **(C)** Western blot showing the levels Syt XI and Sec22b at 2 h post-infection. The % difference in Syt XI levels represents the % difference in band intensities of 2 h-infected vs. NI cells. Band intensities were normalized to β-actin levels. A negative value is indicative of cleavage. Results are representative of two independent experiments.
(TIF)

S8 Fig Flotation of GP63 and PGs in lysates of infected JAWS-II cells. JAWS-II cells transduced with scrambled (shScr) or Sec22b shRNA (shSec22b) were either non-infected or infected with opsonized *L*. *major* Δ*gp63*+*gp63* and Δ*gp63* metacyclic promastigotes for 6 h. A flotation assay was performed where cells were lysed mechanically; sucrose was overlaid over lysates and samples ultracentrifuged for 18 h. **(A)** Western blots show the levels of various *Leishmania* and macrophage proteins in fractionated lysates. GRP78, CNX, CRT, and PDI were used as ER markers; Sec22b as an ERGIC marker; and TCIRG1 as a maker of endosomes and lysosomes. Asterisks (*) indicate non-specific bands of macrophage origin. **(B)** Densitometric analysis of flotation assay shown in (A). To facilitate the analysis of band intensities, heat maps were produced to compare densitometric data in JAWS-II cells transfected with control (shScr) or shRNA to Sec22b (shSec22b). For all studied proteins, including the processed form of GP63, densitometries in Western blots from shSec22b cells were divided by the corresponding densitometries in blots from shScr cells. The log_10_ of the ratios, varying from -1 to 1 were displayed as a colour from green to beige to red. A relative decrease is a negative value, no difference is 0, and an increase is a value greater than 0. The data are representative of two independent experiments. TCL, total cell lysate.
(TIF)

S9 Fig The host cell’s secretory pathway mediates the post-infection egress of GP63 and PGs from the PV. *Leishmania*-containing PVs use the host cell’s ER-ERGIC circuitry to facilitate the redistribution of GP63 and PGs and the ensuing cleavage of GP63 substrates.
(TIF)

S10 Fig Characterization of metacyclic promastigotes. **(A)** Expression of GP63 and LPG in procyclic and metacyclic promastigotes. To compare the expression of GP63 and LPG in different developmental stages of *Leishmania* promastigotes, the lysates of procyclic (early log) and metacyclic promastigotes of the strains used in this study were analyzed. The expression of GP63, LPG and aldolase (ALD) were probed via Western blot and the activity of GP63 was assayed via gelatin zymography. The images shown are representative of two independent experiments. **(B)** Quantification of *L*. *donovani* and *L*. *major* intracellular survival in BMM at 6, 24 and 72 h post-infection. Data are presented as mean ± s.e.m. of n = 3 experiments done in triplicate, with each point representing the number of intracellular parasites found in 100 macrophages.
(TIF)

S11 Fig Immunostaining controls for confocal and electron microscopy. **(A)** To assess the specificity of our co-immunostainings, BMM were infected with opsonized *L*. *major* Δ*gp63*+*gp63* metacyclic promastigotes for 6 h, and prepared for confocal microscopy. Channel cross-talk and secondary antibody background were tested by incubating cells with mismatched antibody combinations, and with secondary antibodies only, respectively. The appropriate combination is at the top leftmost panel. The fluorescence of appropriately matched antibody combinations is depicted in green (αGP63^(mIgG2a)^+αmIgG2a^A488^), red (αLPG^(mIgM)^+αmIgM^A568^) and cyan (αERGIC53^(rIgG)^+αrIgG^A633^); DNA is in blue. White arrowheads denote internalized parasites; bar, 5 μm. **(B)** Immuno-electron microscopy images of representative 6h-infected BMM stained with appropriate (upper leftmost panel) and mismatched antibody combinations. Here, matched secondary antibodies recognize GP63 and Sec23 with gold nanoparticles of size 10 nm and 20 nm, respectively. The red arrowhead denotes a structure where GP63 and Sec23 co-occur; bar, 100nm. ^A^, Alexa dye; α, anti-; m, mouse; r, rabbit; N, BMM nucleus.
(TIF)
