# Supplementary figures and images for: The host cell secretory pathway mediates the export of Leishmania virulence factors out of the parasitophorous vacuole
Source: PLoS Pathog. 2019 Jul 29;15(7):e1007982. doi: 10.1371/journal.ppat.1007982 (PMC6687203; doi:10.1371/journal.ppat.1007982)

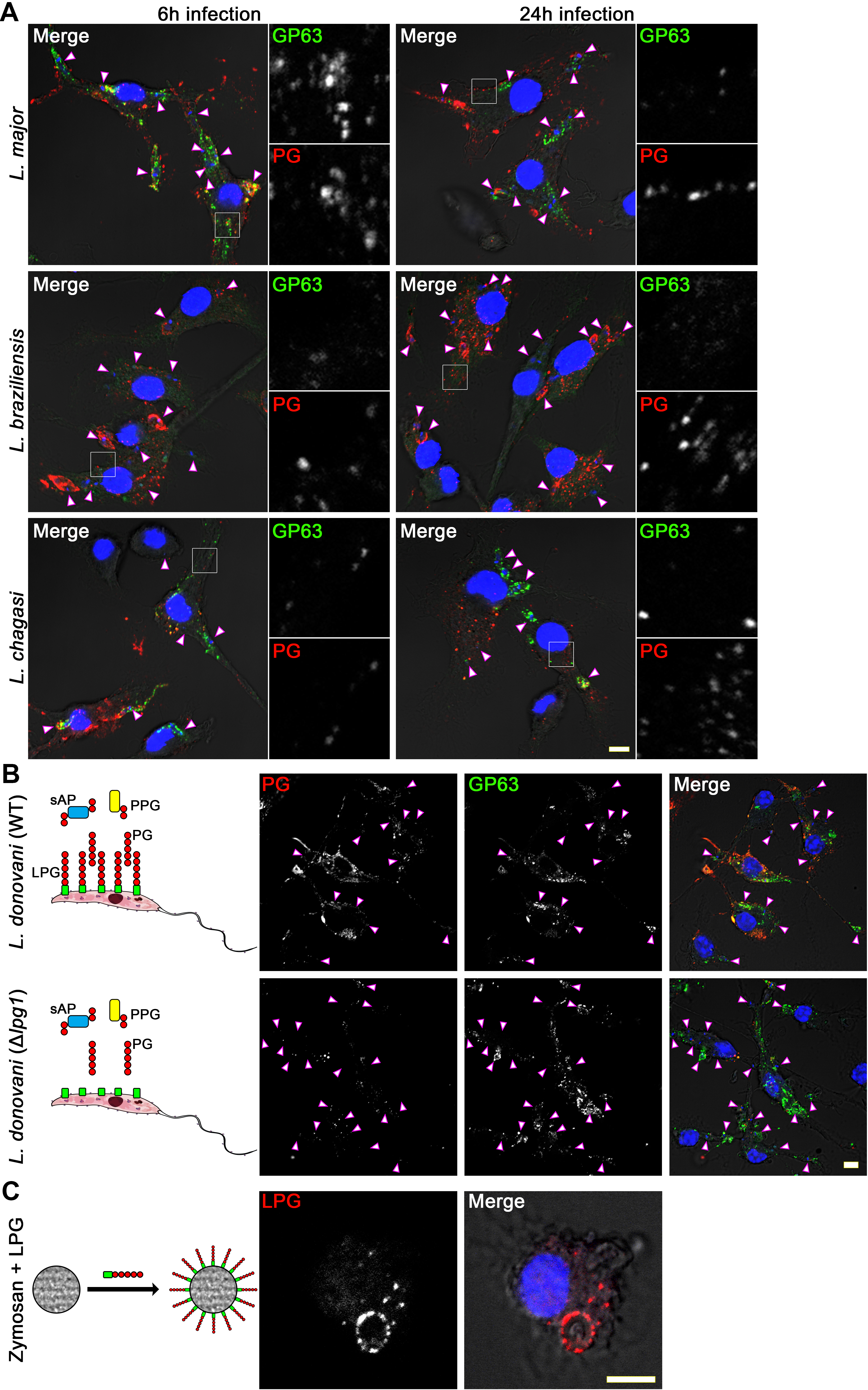

Supplement: S1 Fig — (A) To assess the trafficking and persistence of GP63 and PGs over a period of 6 to 24 h, we infected BMM with opsonized wild type L. major, L. braziliensis, and L. chagasi metacyclic promastigotes. Using immunofluorescence, the redistribution of GP63 and PGs was observed over the indicated time period. 5X-enlarged channel-split insets of representative cytoplasmic regions are shown. (B) To elucidate whether the trafficking of PGs differs in promastigotes that predominantly express the GPI-anchored LPG versus promastigotes that only secrete the repeating disaccharide-phosphate repeats (schema on the left). We infected BMM for 6 h with opsonized wild type and LPG-defective Δlpg1 L. donovani metacyclic promastigotes. (C) To assay whether the redistribution of LPG is a parasite-dependent process, zymosan particles were coated with purified LPG and given to macrophages. Redistribution of LPG was assayed after 1 h via immunofluorescence. LPG or PGs are shown in red, GP63 in green and DNA in blue. Images are representative of two independent experiments and white arrowheads denote internalized parasites. Bar, 5 μm. (TIF) [file ppat.1007982.s001.tif]

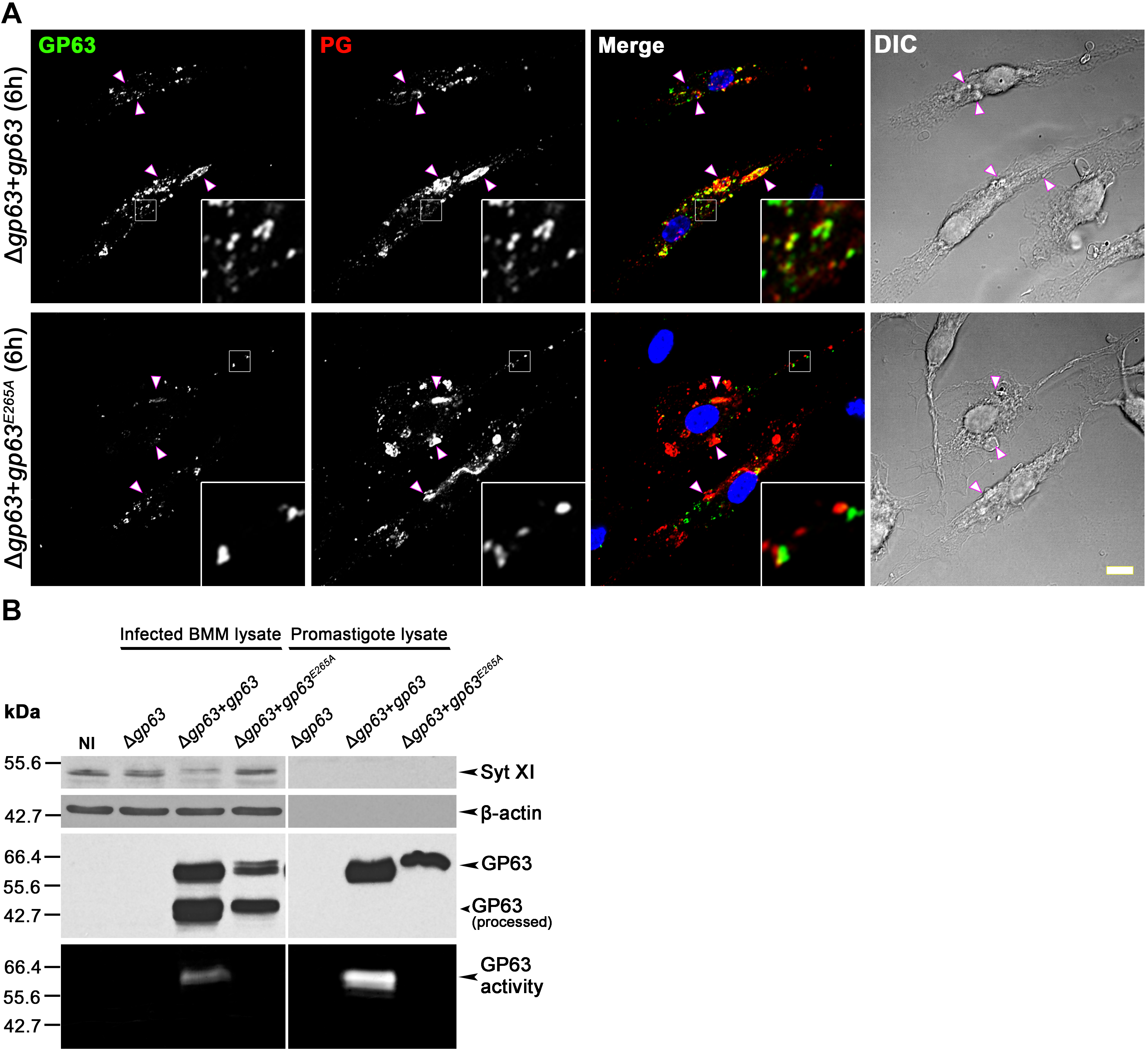

Supplement: S2 Fig — (A) To investigate whether the catalytic activity of GP63 was required for GP63 or PGs to disperse from the PV, we infected BMM with opsonized L. major metacyclic promastigotes expressing catalytically active (Δgp63+gp63) or inactive (Δgp63+gp63E265A) GP63. Six hours post-infection, cells were fixed and prepared for confocal microscopy. GP63 is shown in green, PGs in red and DNA in blue. White arrowheads denote internalized parasites and 5X-enlarged insets are shown. (B) Infected cell lysates and promastigote lysates were probed by Western blot. Murine β-actin was used for loading control. The Syt XI blot and gelatin zymography were used to evaluate GP63 activity. These results are representative of two independent experiments. DIC, differential intensity contrast image; NI, non-infected; bar, 5 μm. (TIF) [file ppat.1007982.s002.tif]

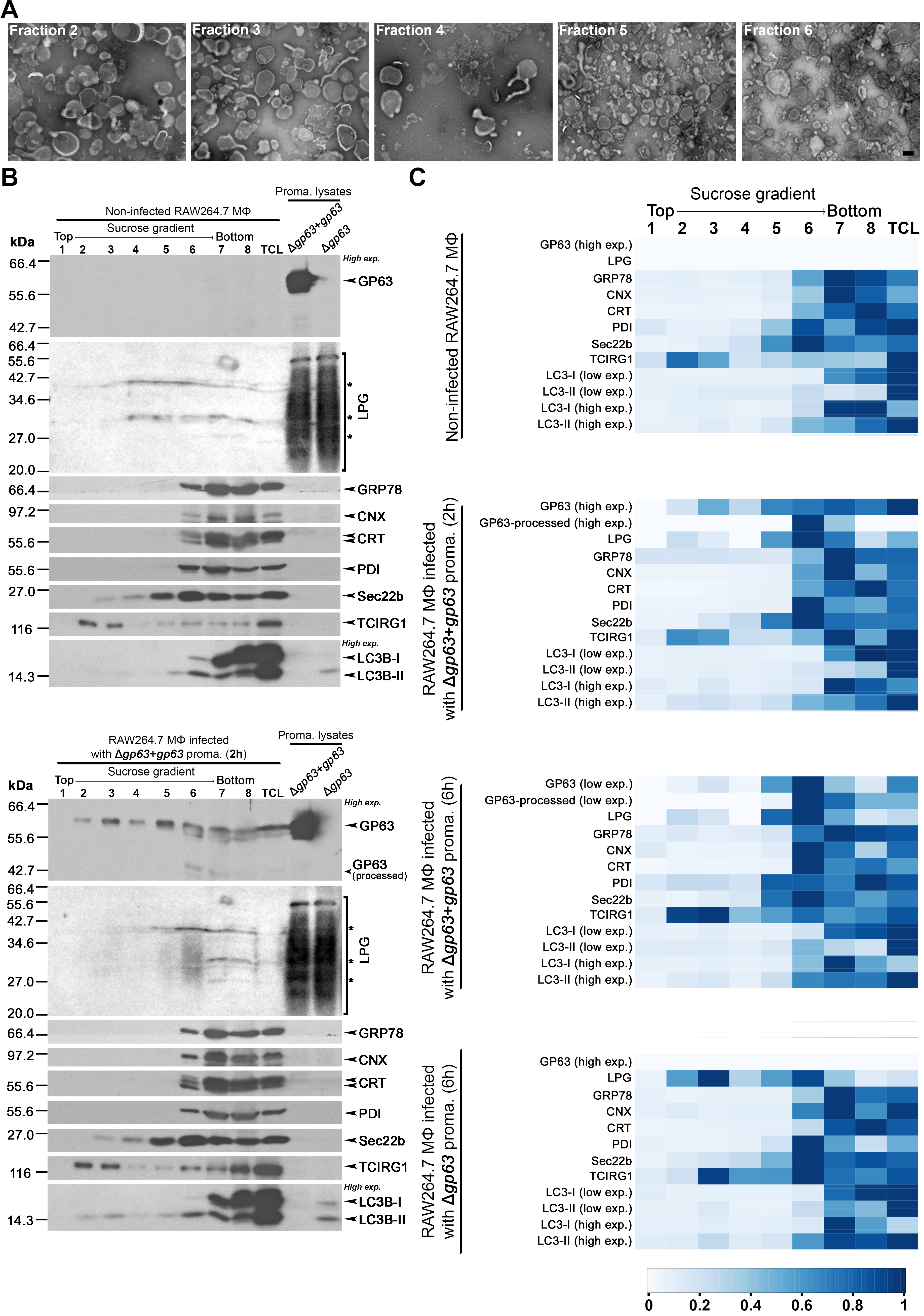

Supplement: S3 Fig — RAW264.7 macrophages were either non-infected or infected with opsonized L. major Δgp63+gp63 metacyclic promastigotes for 2–6 h. A flotation assay was performed where cells were lysed mechanically; sucrose was overlaid over lysates and samples were ultracentrifuged for 18h. Fractions were collected from the top. (A) The presence of vesicles in the collected fractions from 6 h-infected cells (Δgp63+gp63) was verified by electron microscopy and shown here; those from the other conditions were similar (not shown). Bar, 100 nm. (B) Western blots depicting the levels of various Leishmania and macrophage proteins in fractionated lysates from non-infected and 2 h-infected cells; 6 h infections are shown in Fig 4. GRP78, CNX, CRT, and PDI were used as ER markers, Sec22b as an ERGIC marker, and TCIRG1 as a maker of endosomes and lysosomes. Light vesicle-containing fractions are delimited by the exclusive appearance of LC3B-II, which is membrane-bound. The LPG band appears as a smear and asterisks (*) indicate non-specific bands of macrophage origin (see non-infected cell and promastigote lysate lanes). TCL, total cell lysate. (C) Densitometric analysis of flotation assay in Fig 4A and S3B Fig. To facilitate the comparison of band intensities in each condition, heat maps were produced from densitometry data. For each protein (e.g., Sec22b in non-infected cells), the band with the highest intensity was assigned a value of 1, and the other intensities in that group (fraction 1 to TCL) were normalized with respect to that band. Since there is no GP63 in non-infected cells, background from this condition was subtracted from the other conditions (infected cells). Densitometries were then normalized as above. The densitometry of the ~42 kDa fragment (GP63-processed) was also analyzed. In the case of LPG, a box encasing the smears was used to calculate the densitometries. Since there are no PGs in non-infected cells, background from this condition, including that given by the non-sp [file ppat.1007982.s003.tif]

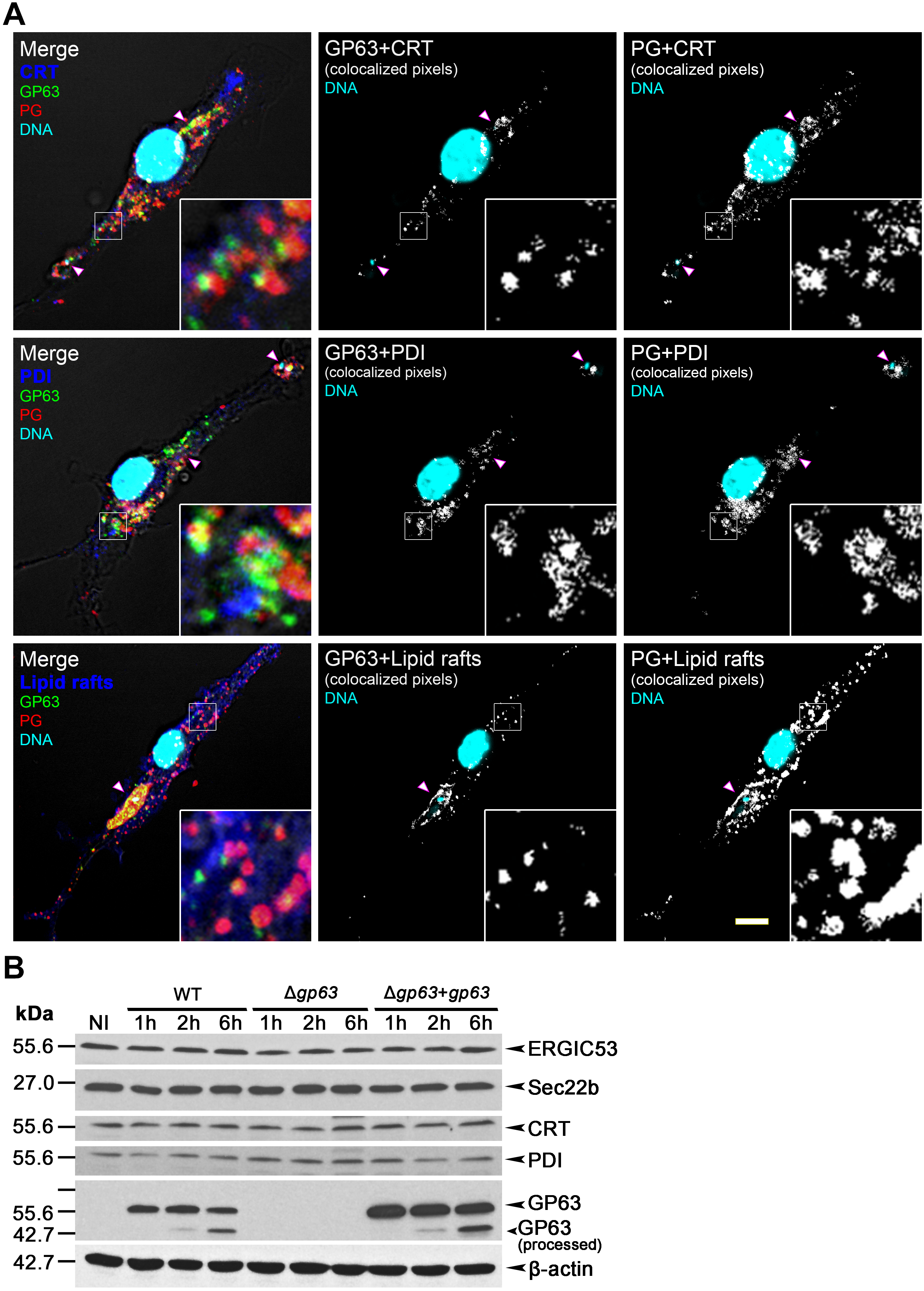

Supplement: S4 Fig — (A) BMM were infected with opsonized L. major Δgp63+gp63 metacyclic promastigotes for 6h and the colocalization (white pixels, middle and rightmost panels) of GP63 (green) or PGs (red) with ER markers (blue) CRT and PDI was assessed by confocal immunofluorescence microscopy. DNA is in cyan. 5X-enlarged insets of representative cytoplasmic regions are shown. White arrowheads denote internalized parasites. Bar, 5 μm. (B) GP63 does not cleave resident ER and ERGIC proteins. To investigate whether ER and ERGIC proteins are cleaved by GP63, BMM were infected with opsonized L. major WT, Δgp63 or Δgp63+gp63 metacyclic promastigotes. The integrity of the various ER and ERGIC markers was assayed by Western blot. Results are representative of at least two independent experiments. NI, non-infected. (TIF) [file ppat.1007982.s004.tif]

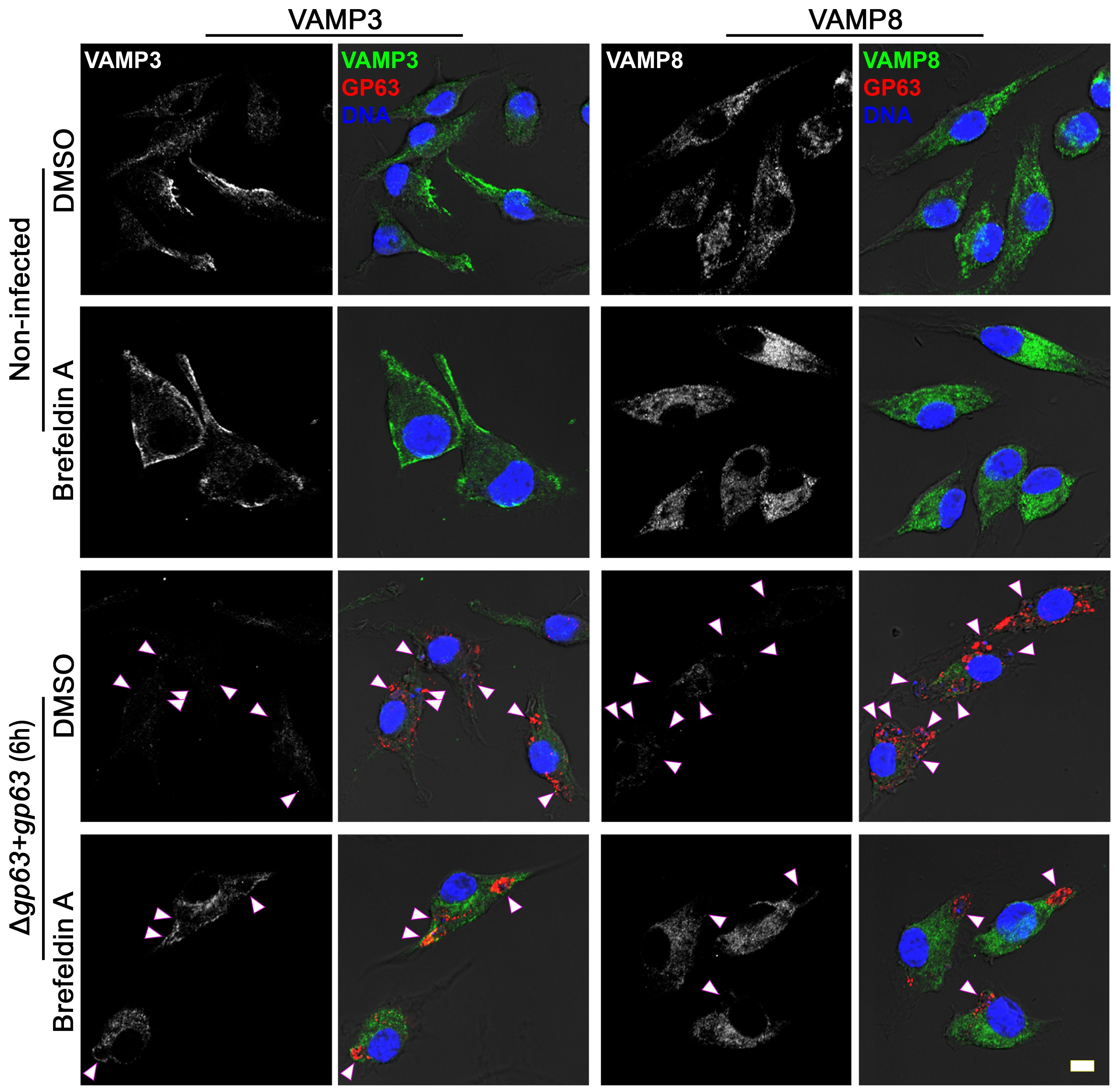

Supplement: S5 Fig — BMM were treated with brefeldin A or DMSO prior to infection with opsonized L. major Δgp63+gp63 metacyclic promastigotes for 6h. The impact of these treatments on the degradation of VAMP3 and VAMP8 (green) by GP63 (red) was assayed via immunofluorescence. White arrowheads denote internalized parasites and DNA is in blue. Bar, 5 μm. (TIF) [file ppat.1007982.s005.tif]

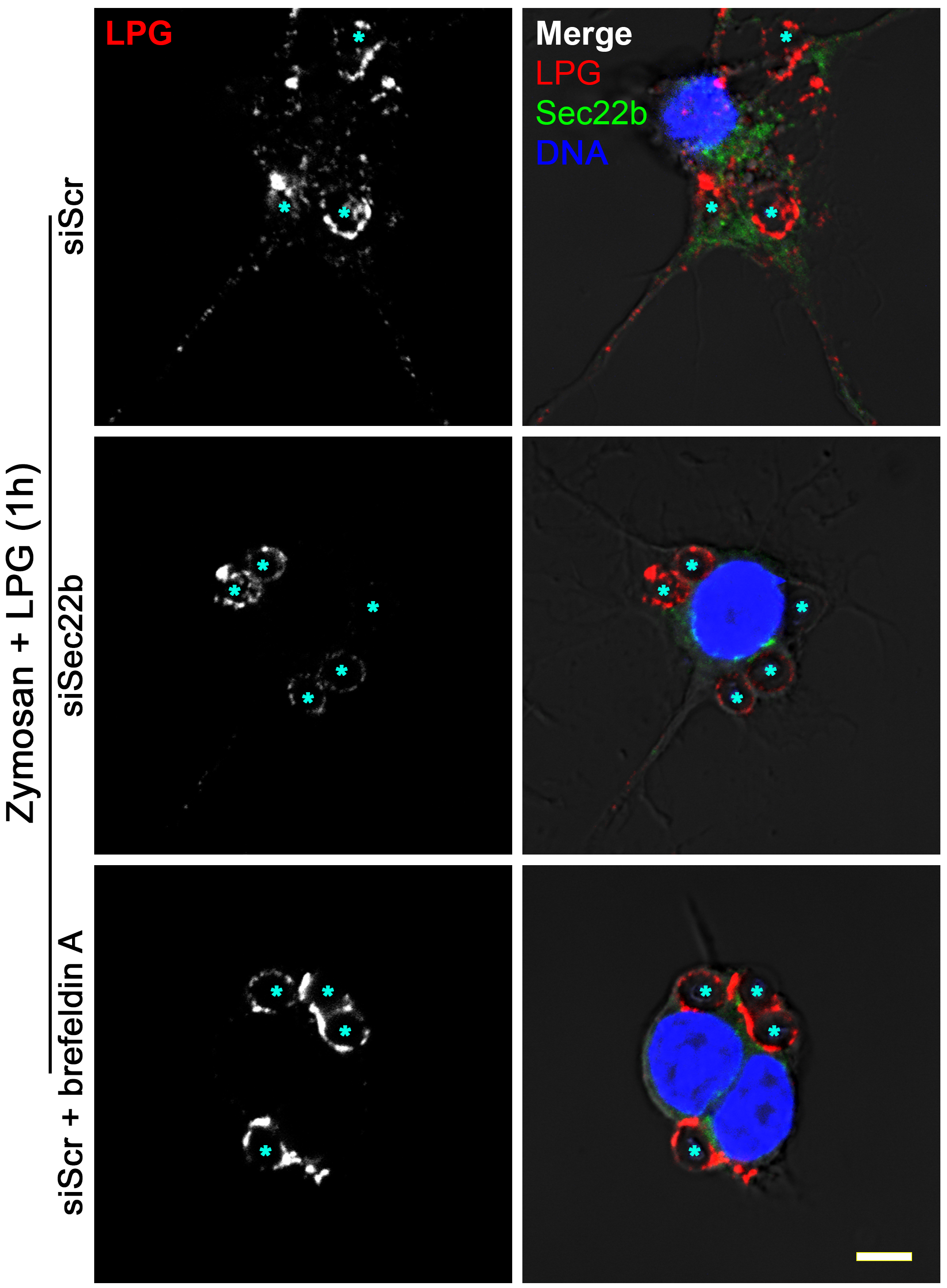

Supplement: S6 Fig — To assay whether the redistribution of LPG is a host cell-dependent process, zymosan particles were coated with purified LPG and given to RAW264.7 macrophages transfected with siRNA or treated with brefeldin A. Redistribution of LPG (red) was assayed after 1 h via immunofluorescence. Sec22b is in green, DNA in blue, and the position of zymosan particles is denoted by an asterisk. Images are representative of two independent experiments; bar, 5 μm. (TIF) [file ppat.1007982.s006.tif]

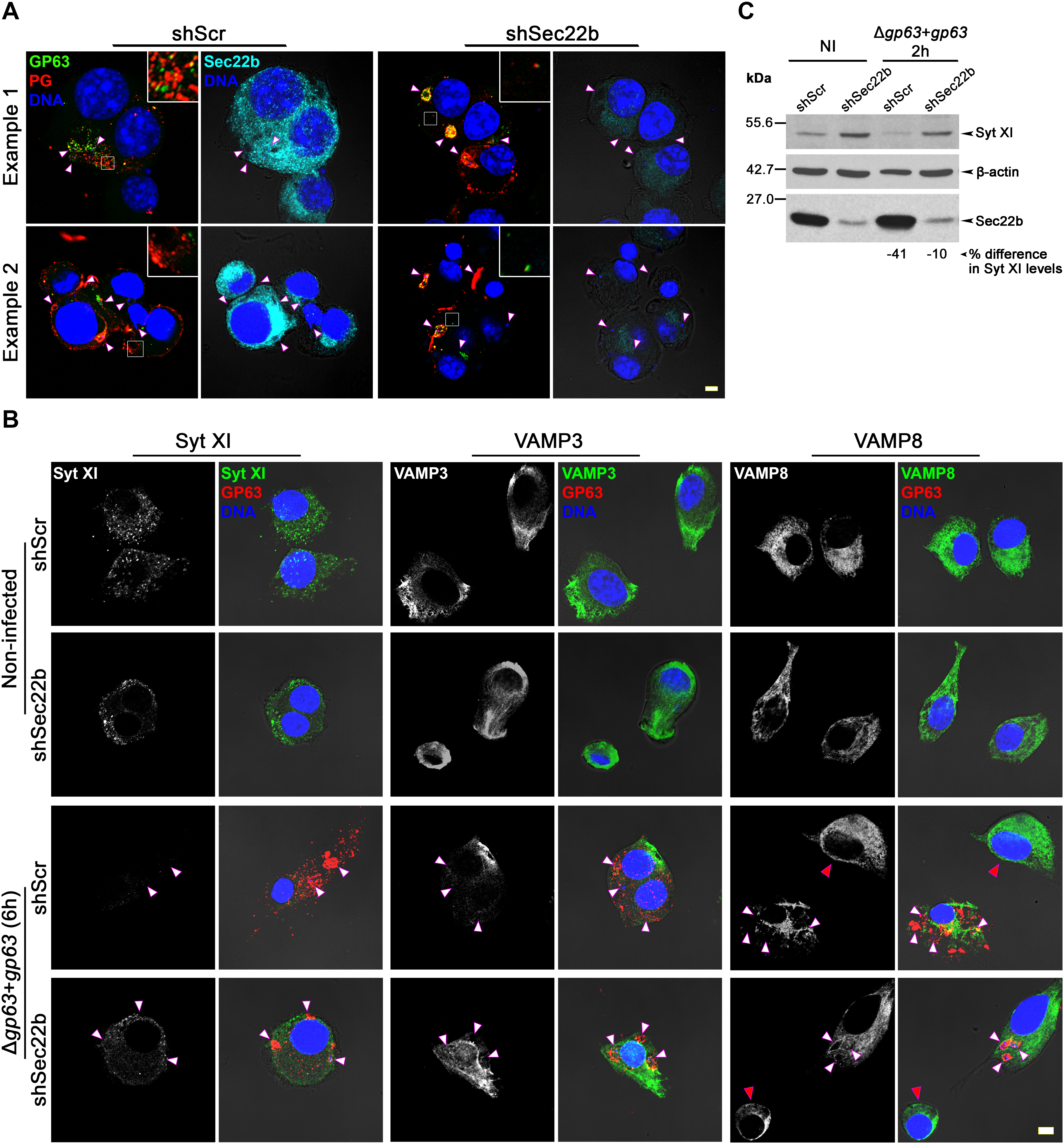

Supplement: S7 Fig — (A) JAWS-II cells transduced with scrambled (shScr) or Sec22b shRNA (shSec22b) were infected with opsonized L. major Δgp63+gp63 metacyclic promastigotes for 6 h. The effect of Sec22 (cyan) KD on the redistribution of GP63 (green) and PGs (red) was visualized. 5X-enlarged insets of representative cytoplasmic regions are shown. (B) In Sec22b-KD JAWS-II cells, the degradation of Syt XI, VAMP3 and VAMP8 (green) by GP63 (red) was assayed via immunofluorescence. In panels (A) and (B), white and red arrowheads denote internalized parasites and non-infected cells, respectively. DNA is in blue; bar, 5 μm. (C) Western blot showing the levels Syt XI and Sec22b at 2 h post-infection. The % difference in Syt XI levels represents the % difference in band intensities of 2 h-infected vs. NI cells. Band intensities were normalized to β-actin levels. A negative value is indicative of cleavage. Results are representative of two independent experiments. (TIF) [file ppat.1007982.s007.tif]

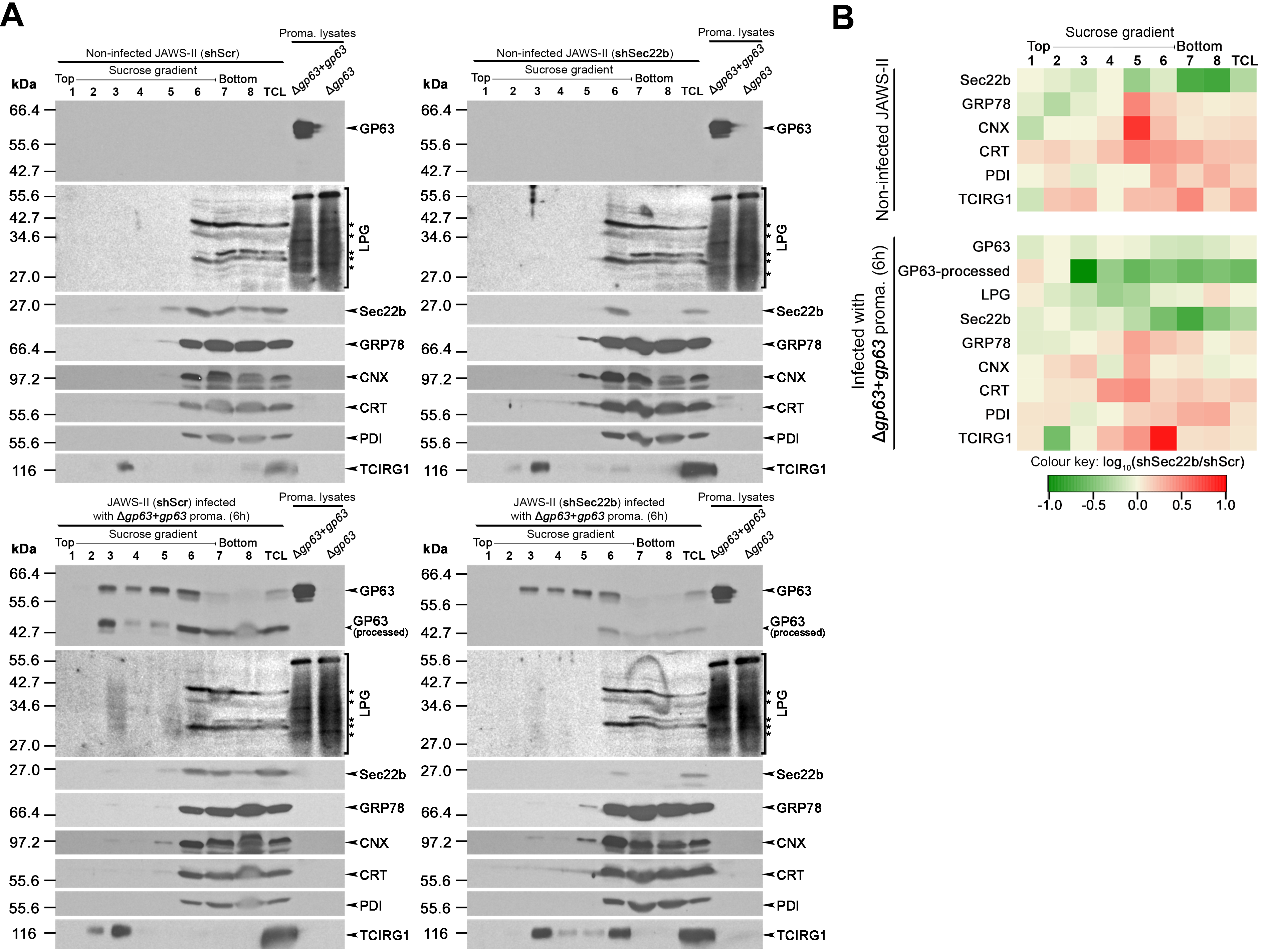

Supplement: S8 Fig — JAWS-II cells transduced with scrambled (shScr) or Sec22b shRNA (shSec22b) were either non-infected or infected with opsonized L. major Δgp63+gp63 and Δgp63 metacyclic promastigotes for 6 h. A flotation assay was performed where cells were lysed mechanically; sucrose was overlaid over lysates and samples ultracentrifuged for 18 h. (A) Western blots show the levels of various Leishmania and macrophage proteins in fractionated lysates. GRP78, CNX, CRT, and PDI were used as ER markers; Sec22b as an ERGIC marker; and TCIRG1 as a maker of endosomes and lysosomes. Asterisks (*) indicate non-specific bands of macrophage origin. (B) Densitometric analysis of flotation assay shown in (A). To facilitate the analysis of band intensities, heat maps were produced to compare densitometric data in JAWS-II cells transfected with control (shScr) or shRNA to Sec22b (shSec22b). For all studied proteins, including the processed form of GP63, densitometries in Western blots from shSec22b cells were divided by the corresponding densitometries in blots from shScr cells. The log10 of the ratios, varying from -1 to 1 were displayed as a colour from green to beige to red. A relative decrease is a negative value, no difference is 0, and an increase is a value greater than 0. The data are representative of two independent experiments. TCL, total cell lysate. (TIF) [file ppat.1007982.s008.tif]

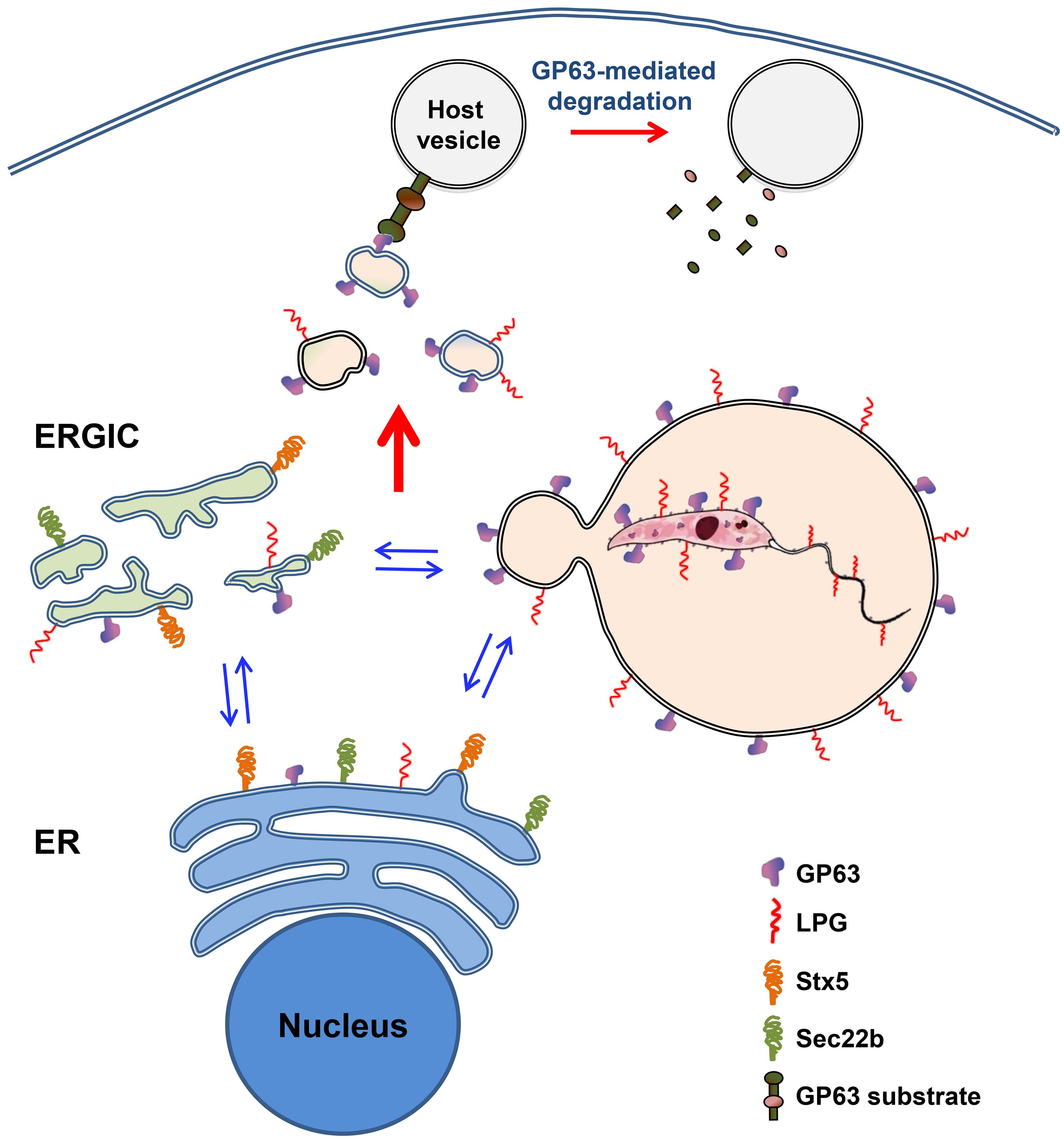

Supplement: S9 Fig — Leishmania-containing PVs use the host cell’s ER-ERGIC circuitry to facilitate the redistribution of GP63 and PGs and the ensuing cleavage of GP63 substrates. (TIF) [file ppat.1007982.s009.tif]

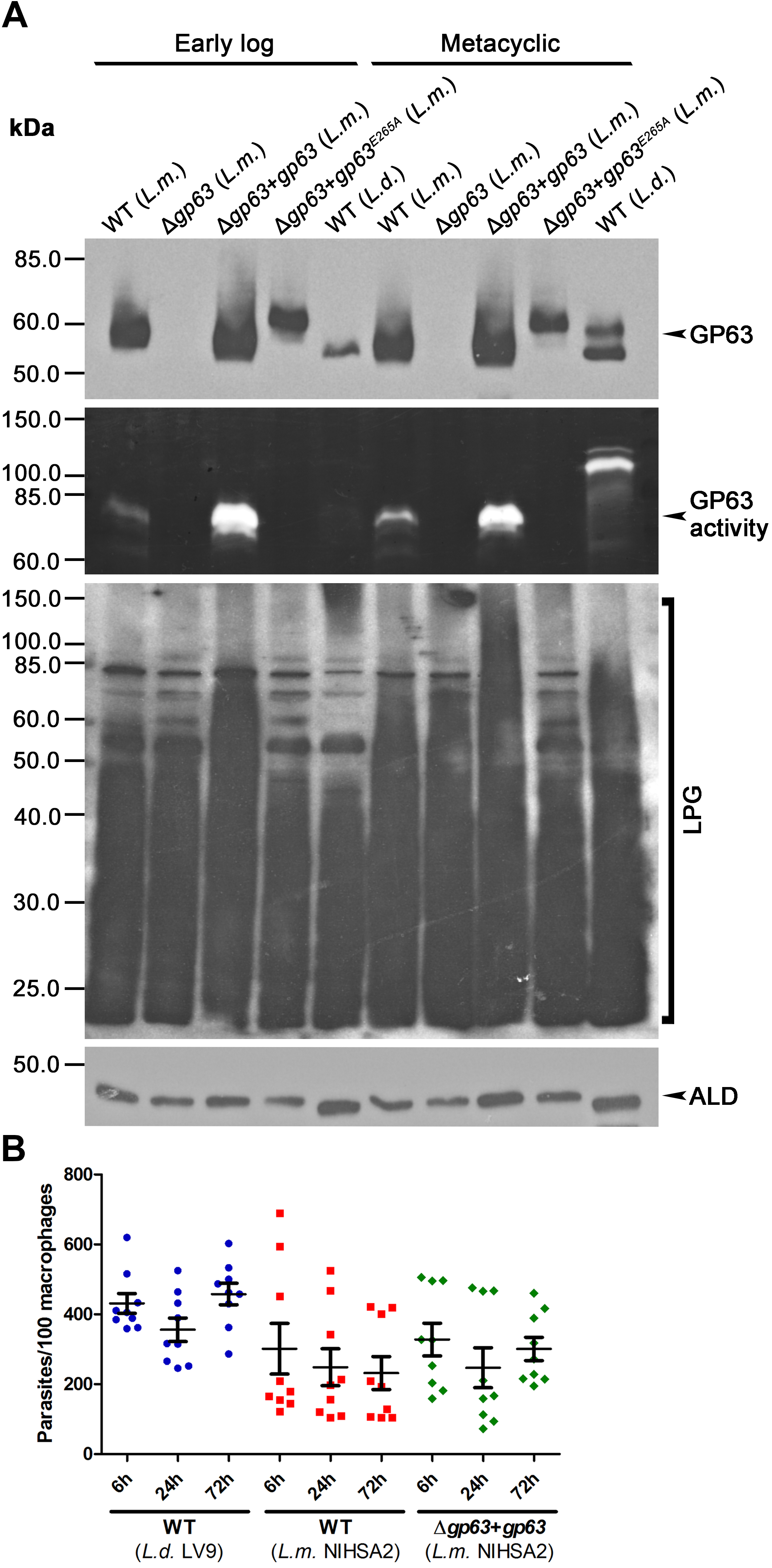

Supplement: S10 Fig — (A) Expression of GP63 and LPG in procyclic and metacyclic promastigotes. To compare the expression of GP63 and LPG in different developmental stages of Leishmania promastigotes, the lysates of procyclic (early log) and metacyclic promastigotes of the strains used in this study were analyzed. The expression of GP63, LPG and aldolase (ALD) were probed via Western blot and the activity of GP63 was assayed via gelatin zymography. The images shown are representative of two independent experiments. (B) Quantification of L. donovani and L. major intracellular survival in BMM at 6, 24 and 72 h post-infection. Data are presented as mean ± s.e.m. of n = 3 experiments done in triplicate, with each point representing the number of intracellular parasites found in 100 macrophages. (TIF) [file ppat.1007982.s010.tif]

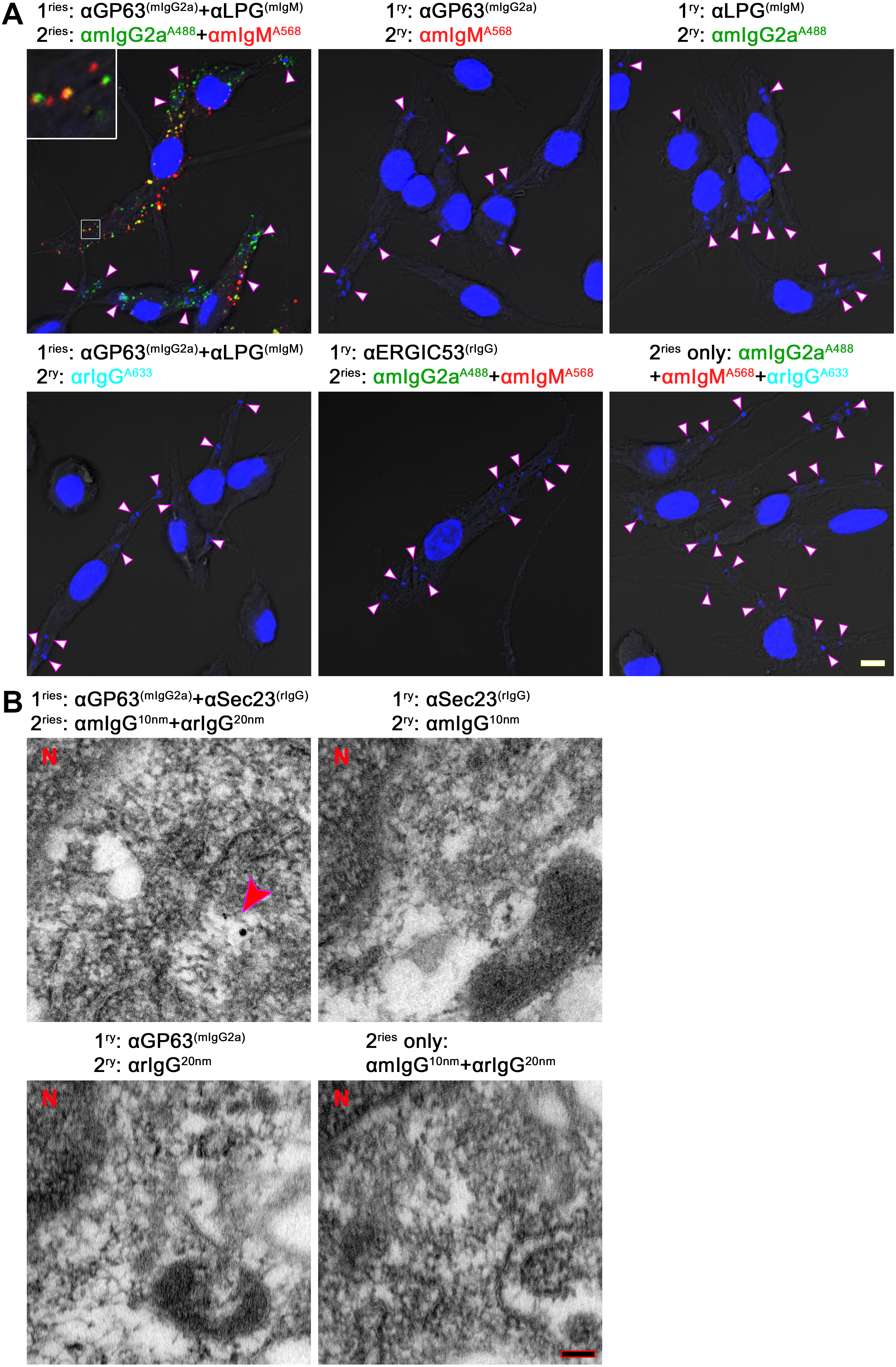

Supplement: S11 Fig — (A) To assess the specificity of our co-immunostainings, BMM were infected with opsonized L. major Δgp63+gp63 metacyclic promastigotes for 6 h, and prepared for confocal microscopy. Channel cross-talk and secondary antibody background were tested by incubating cells with mismatched antibody combinations, and with secondary antibodies only, respectively. The appropriate combination is at the top leftmost panel. The fluorescence of appropriately matched antibody combinations is depicted in green (αGP63(mIgG2a)+αmIgG2aA488), red (αLPG(mIgM)+αmIgMA568) and cyan (αERGIC53(rIgG)+αrIgGA633); DNA is in blue. White arrowheads denote internalized parasites; bar, 5 μm. (B) Immuno-electron microscopy images of representative 6h-infected BMM stained with appropriate (upper leftmost panel) and mismatched antibody combinations. Here, matched secondary antibodies recognize GP63 and Sec23 with gold nanoparticles of size 10 nm and 20 nm, respectively. The red arrowhead denotes a structure where GP63 and Sec23 co-occur; bar, 100nm. A, Alexa dye; α, anti-; m, mouse; r, rabbit; N, BMM nucleus. (TIF) [file ppat.1007982.s011.tif]
